# Induced Autoimmunity against Gonadal Proteins Affects Gonadal Development in Juvenile Zebrafish

**DOI:** 10.1371/journal.pone.0114209

**Published:** 2014-12-01

**Authors:** Christopher Presslauer, Kazue Nagasawa, Dalia Dahle, Joanna Babiak, Jorge M. O. Fernandes, Igor Babiak

**Affiliations:** Faculty of Biosciences and Aquaculture, University of Nordland, 8049 Bodø, Norway; Leibniz Institute for Age Research - Fritz Lipmann Institute (FLI), Germany

## Abstract

A method to mitigate or possibly eliminate reproduction in farmed fish is highly demanded. The existing approaches have certain applicative limitations. So far, no immunization strategies affecting gonadal development in juvenile animals have been developed. We hypothesized that autoimmune mechanisms, occurring spontaneously in a number of diseases, could be induced by targeted immunization. We have asked whether the immunization against specific targets in a juvenile zebrafish gonad will produce an autoimmune response, and, consequently, disturbance in gonadal development. Gonadal soma-derived factor (Gsdf), growth differentiation factor (Gdf9), and lymphocyte antigen 75 (Cd205/Ly75), all essential for early gonad development, were targeted with 5 immunization tests. Zebrafish (n = 329) were injected at 6 weeks post fertilization, a booster injection was applied 15 days later, and fish were sampled at 30 days. We localized transcripts encoding targeted proteins by *in situ* hybridization, quantified expression of immune-, apoptosis-, and gonad-related genes with quantitative real-time PCR, and performed gonadal histology and whole-mount immunohistochemistry for Bcl2-interacting-killer (Bik) pro-apoptotic protein. The treatments resulted in an autoimmune reaction, gonad developmental retardation, intensive apoptosis, cell atresia, and disturbed transcript production. Testes were remarkably underdeveloped after anti-Gsdf treatments. Anti-Gdf9 treatments promoted apoptosis in testes and abnormal development of ovaries. Anti-Cd205 treatment stimulated a strong immune response in both sexes, resulting in oocyte atresia and strong apoptosis in supporting somatic cells. The effect of immunization was FSH-independent. Furthermore, immunization against germ cell proteins disturbed somatic supporting cell development. This is the first report to demonstrate that targeted autoimmunity can disturb gonadal development in a juvenile fish. It shows a straightforward potential to develop auto-immunization-based technologies to mitigate fish reproduction before they reach maturation. However, the highly variable results between treatments and individuals suggest significant optimization should be performed to achieve the full potential of this technology.

## Introduction

Control of reproduction in farmed, pest, and pet animals is an important issue with multiple implications in economy, environment, and welfare [1],[2],[3]. Unwanted sexual maturation of fish before they reach market size remains a significant constraint in aquaculture. For example, in farmed Atlantic cod (*Gadus morhua*), maturation often occurs in the second year, while in Atlantic salmon (*Salmo salar*) it can happen after just one winter in seawater. Both cases result in loss of growth, condition, and flesh quality [4], [5], [6]. In addition to economic consequences, precocious sexual maturation of farmed fish creates a threat of genetic pollution to natural populations because of escapes and uncontrolled gamete production [7]. The creation of mono-sex stocks can mitigate some concerns regarding gamete production and growth in some species such as catfish (*Pelteobagrus spp.*) or tilapia (*Oreochromis spp.*) [8], [9]. However, it does not resolve concerns about reproductive potential of escapees and flesh quality of farmed fish.

Therefore, a method to mitigate or possibly eliminate gonadal growth in farmed fish is highly demanded. Transgenic [10], morpholino-based knockdown [11], or induced triploidization [12] approaches, resulting in partial or complete gamete ablation and consequently in reproduction disability, have their apparent limitations related to welfare, food safety, costs, or application potential to mass-scale production. A method based on intrinsic properties of the immune system, which does not require genetic modifications, is cost-effective and applicable at the industrial production level, would overcome the aforementioned limitations.

The majority of efforts in immunocontraception and immunosterilization have been focused on the mammalian reproductive system with a purpose to control wildlife populations [3], [13], [14], [15]. Usually the treatments stimulate the production of autoimmune antibodies to either bind to the gametes directly and prevent their function, or interfere with the GnRH pathway to prevent gamete production; some mammalian vaccines have resulted in significantly impaired fertility [14]. While immunocontraception serves the purpose of preventing outbreak of mammalian pest populations, it is not suitable for aquaculture as it does not affect gonadal development and the associated deteriorated growth and flesh quality. A study on rainbow trout (*Oncorhynchus mykiss*) demonstrated that injection of a gonadal extract resulted in a limited autoimmune response in gonads [16]. However, that study was performed on few individuals only and no recent published studies have explored this concept further.

In the current study, we have asked whether the vaccination against specific targets in a juvenile gonad will produce an autoimmune response, and consequently, if it will disturb gonadal development. We chose zebrafish (*Danio rerio*) as a model. Zebrafish undergo a juvenile hermaphroditism sex differentiation pattern [17], [18], [19]. The gonad first develops perinucleolar oocytes around 4 weeks post-fertilization (pf). While the timing of sexual differentiation can vary, a minimum length of 8 mm is a pre-requisite for development beyond an undifferentiated gonad [17]. Then either ovary is developed, or the oocytes degenerate and the gonad transforms into testis, with the first spermatocytes observed around 7 weeks pf. Completion of sexual differentiation occurs by weeks 11–12 pf [17]. However, the timing of this process is widely variable between individuals, as well as depending on fish strain and conditions.

After preliminary studies, we selected three proteins essential for gonad development as potential targets for induced autoimmunity: gonadal soma-derived factor (Gsdf), growth differentiation factor 9 (Gdf9), and lymphocyte antigen 75 (Cd205/Ly75). Gsdf is a member of TGFβ superfamily expressed in supporting somatic cells of ovary and testis, granulosa and Sertoli cells, respectively [20]. *Gsdf* expression in both sexes is highest when germ cells are in the spermatogonial or oogonial stages of gametogenesis, while expression decreases when germ cells enter the meiotic and postmeiotic stages [20]. This mechanism appears to be conserved in teleosts, as it is similar in rainbow trout, medaka (*Oryzias latipes*), and zebrafish [20], [21], [22], [23]. In rainbow trout, it was demonstrated that *gsdf* knock-down during embryogenesis suppressed germ cell populations, while recombinant Gsdf was used for dose-dependent proliferation of type A spermatogonia in testis cell culture [21]. It has been suggested that since oocytes do not proliferate under this treatment, *gsdf* may have a different function in ovaries than testes.

Gdf9 is another member of the TGFβ superfamily secreted from germ cells in both sexes and is involved in follicle development [24], [25], [26], [27], [28]. In *gdf9* knock-out mice, folliculogenesis stops at the primary follicle stage [29]. In male cats, *gdf9* is expressed specifically within the germ cell lineage and is involved in signaling to Sertoli and Leydig cells to regulate tight junctions [28]. In zebrafish, *gdf9* is expressed predominantly in testes [30], but also plays a role in ovarian follicle development, where it regulates tight junction integrity between oocytes and follicle cells [26].

Lastly, Cd205 is an endocytic type I C-type lectin-like receptor belonging to the macrophage mannose receptor (MMR) family and consisting of a single polypeptide chain. In mammals, *Cd205* is strongly expressed in dendritic cells, where the protein acts as an endocytic receptor, binding with antigens and carrying them through the MHC class I and II pathways to present them to CD4+ and CD8+ T cells [31], [32], [33]. In fish, *cd205* is expressed in multiple tissues. However, within the gonad it is expressed exclusively in the germline [34], [35]. In rainbow trout, the protein was localized to the surface of early germ cells, mainly primordial germ cells, type A spermatogonia, oogonia, and cortical alveoli oocytes. It was not found in spermatocytes, spermatids, spermatozoa, and vitellogenic oocytes [34]. While its function in teleost germ cells is unknown, the protein likely maintains a role in antigen presentation and related immune function.

In the current study, we immunized 6 week-old juvenile zebrafish against Gsdf, Gdf9, or Cd205 during early sexual development in order to induce autoimmune response in gonads. We gave a booster injection 15 days later, and sampled the fish at both 15 and 30 days after the initial treatment. We used *in situ* hybridization to localize transcripts encoding the target proteins. We performed gonadal histology, quantitative real-time PCR (qPCR) of immune-, apoptosis-, and gonad development-related genes, as well as whole-mount immunohistochemistry for the pro-apoptotic protein Bcl2-interacting-killer (Bik). We have demonstrated that gonadal development in juvenile zebrafish was affected *via* autoimmune mechanism.

## Results

### Localization of *gsdf*, *gdf9*, and *cd205* transcripts within the gonad

**Figure 1:**
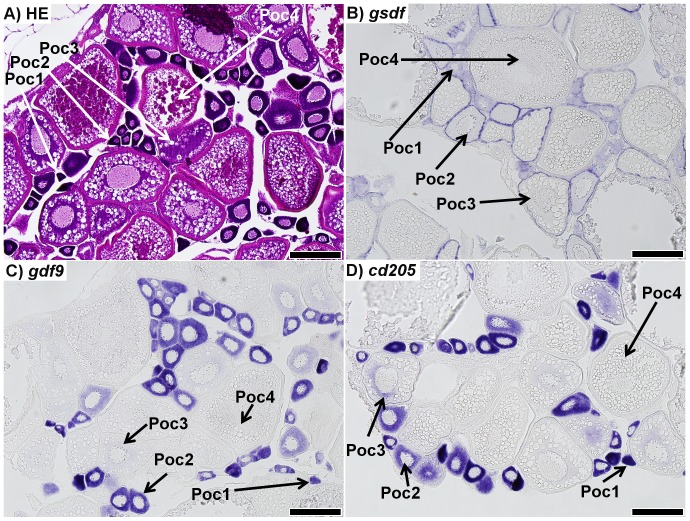
Sections of ovaries from 7 month-old zebrafish. A) Representative haematoxylin-eosin (HE) staining of the mature zebrafish ovary. B) *in situ* hybridization (ISH) of *gsdf* transcripts in the ovary; staining shows *gsdf* transcripts are localized in somatic cells surrounding primary oocytes. The staining is strongest in stage 2 primary oocytes (Poc2) and previtellogenic oocytes (Poc3). C) ISH of *gdf9* transcripts in the ovary; strong signal is detected in stage 1 and 2 primary oocytes (Poc1 and Poc2). Weak signal is detected in previtellogenic oocytes (Poc3), while no signal was detected in vitellogenic oocytes (Poc4). D) ISH of *cd205* transcripts in the ovary; strong signal is detected in stage 1 and 2 primary oocytes, with weak signal in previtellogenic oocytes, and no transcript detected in vitellogenic oocytes. All scalebars represent 200 µm.

*In situ* hybridization (ISH) targeting transcripts of *gsdf*, *gdf9*, and *cd205* was performed on sections of 7 month-old zebrafish gonads. As a reference for normal gonadal development, hematoxylin-eosin (HE) staining was also performed. The zebrafish ovary was composed of germ cells at various stages of developmental advancement (Fig. 1A). ISH of *gsdf* showed the transcripts were localized in supporting somatic cells surrounding stage 1 and stage 2 primary oocytes, as well as larger previtellogenic oocytes (Fig. 1B). No signal was detected around vitellogenic oocytes. ISH for *gdf9* and *cd205* showed the same expression pattern (Fig. 1C, D).

**Figure 2:**
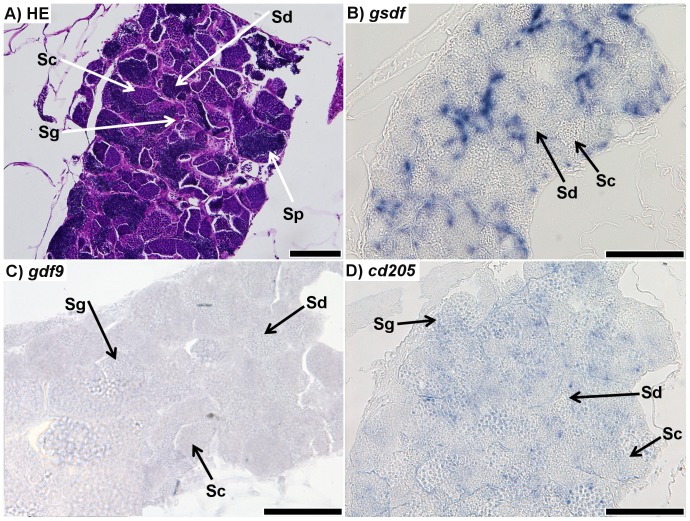
Sections of testis from 7 month-old zebrafish. A) Respresentative haematoxylin-eosin (HE) staining of the mature zebrafish testis; spermatogonia (Sg), spermatocytes (Sc), spermatids (Sd), and spermatozoa (Sp) could all be positively identified. B) *in situ* hybridization (ISH) of *gsdf* transcripts in the testis; staining shows strong signal throughout the testis, although it is unclear from which cell type it originates; no staining is visible in spermatocytes or spermatids. Spermatogonia and spermatozoa could not be identified. C) ISH of *gdf9* transcripts in testis; faint signal was found throughout the testis. It is unclear which cell types the signal is localized to. D) ISH of *cd205* transcripts in the testis; the signal is strongest in spermatogonia, while it is not visible in spermatocytes or spermatids. Spermatozoa could not be identified. All scalebars represent 100 µm.

The mature zebrafish testis was composed of clusters of germ cells at various stages of spermatogenesis (Fig. 2A). *gsdf* transcripts showed strong signal in cells surrounding germ cell clusters (Fig. 2B). Although the cell type from which the signal originated was not determined, no signal in spermatocytes, spermatids, or spermatozoa was observed. Very weak signal was detected for *gdf9* ISH. It was not certain what cell types expressed *gdf9* transcripts (Fig. 2C). ISH for *cd205* resulted in a comparatively weak signal, which appeared to originate from spermatogonia, but not spermatocytes, spermatids or sperm (Fig. 2D).

### Preliminary trial: effect of autoimmunization on adult female growth

To determine whether immunization against gonadal targets influences gonad weight and overall growth of zebrafish, a preliminary trial was performed on adult zebrafish females. Fish were immunized against four different proteins: Zona pellucida C (Zpc), Cd205, Insulin-like growth factor 3 (Igf3), or Riboflavin carrier protein (Rcp). At 10 days post-treatment (10 dpt), significant effect of treatment on fish weight (*p* = 0.004) and gonadosomatic index, GSI% (*p*<0.001) was found. Fish from anti-Zpc and anti-Cd205 treatments had significantly lower weight compared to controls; the length of treated fish was not different from controls (Table S1). The reduction in total weight in anti-Zpc and anti-Cd205 treatments likely resulted from a loss in gonadal weight, because the GSI% of fish in both treatments was significantly lower than in controls.

At 20 dpt, no significant differences in weight and length of fish were found. The effect of treatment on GSI% was significant (*p*<0.001). Fish from anti-Zpc variant had significantly lower GSI% than controls. The GSI% of fish from anti-Zpc and anti-Cd205 treatments was significantly lower than in PBS-injected controls. Other treatments did not differ from either non-injected or PBS-injected controls (Table S1).

At 30 dpt, significant effects of treatment on fish weight (*p* = 0.003) and GSI% (*p*<0.001) were observed. Fish from the anti-Zpc and anti-Igf3 treatments were significantly heavier than control fishes; however, their GSI% did not differ significantly from control fish (Table S1). The GSI% of fish treated with anti-Cd205 was significantly lower than in any other treatment or in control fish.

### Effect of treatment on juvenile zebrafish growth

**Figure 3:**
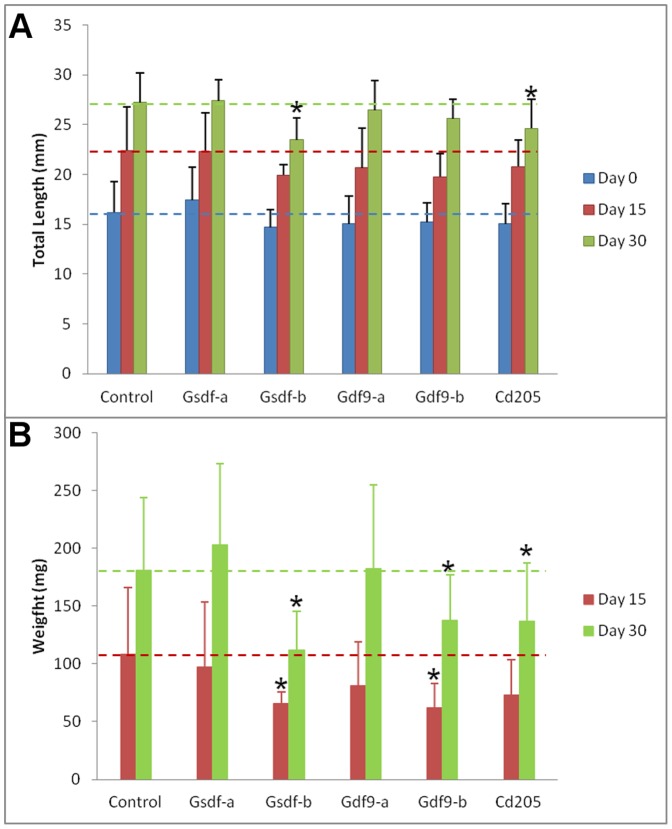
Total length (A) and weight (B) of fish over the course of the experiment. Weight at the start of treatment (day 0) was not measured. Stars represent a statistically significant difference from the control group (ANOVA, LSD test at *p*<0.05) at the given time point. Error bars represent standard deviation.

In the main experiment, five treatments against three gonadal proteins were tested: anti-Gsdf (two treatments, -a and –b), anti-Gdf9 (two treatments, -a and –b), and anti-Cd205. Fish were measured for total length prior to the first injection (day 0) and at 15 and 30 dpt, and weighted at 15 and 30 dpt. Length of fish at the day 0 was homogenous among treatment groups (*p*>0.05). At 15 dpt, fish from the anti-Gsdf-b treatment had significantly lower weight than controls, but no significant differences in length were recorded. At 30 dpt, more distinct retardation in growth was observed: fish from 3 out of 5 treatment variants had significantly lower weight than control fish; of them, fish from two variants (anti-Gsdf-b and anti-Cd205) were significantly shorter than control fish (Fig. 3).

### Sexually dimorphic gene expression

Total RNA was extracted from individuals sampled at both 15 and 30 dpt. Quantitative real-time PCR was then performed to compare relative expression of eight immune-, apoptosis, and gonadal-related genes. All investigated genes showed highly significant sexually dimorphic expression in juvenile zebrafish gonads (*p*<0.001). Expression of *T-cell receptor alpha constant* (*tcrac*), *vasa*, and *cytochrome P450 gonadal isoform* (*cyp19a1a*) was female-enhanced, whereas expression of *immunoglobulin M heavy chain secretory form* (*igμ*), *bcl-2-interacting-killer* (*bik*), *gsdf*, *inhibin alpha subunit* (*inhα*), and *anti-Müllerian hormone* (*amh*) was male-enhanced (Table 1 and Table S2). Correlations between transcript relative abundances followed this pattern: testis-enhanced *bik*, *gsdf*, *inhα*, and *amh* showed high and positive correlation with each other in both control and treated groups, while *igμ* transcript level showed high and positive correlation with *bik, gsdf*, and *amh* (Table 2). Ovary-enhanced *tcrac, vasa*, and *cyp19a1a* transcript levels correlated highly and positively with each other (Table 2). Therefore, analysis of the effect of autoimmunization on gene expression was conducted on males and females separately.

**Table 1:** Sex-dimorphic gene expression in juvenile zebrafish gonads.

	Female		Male		Fold Change
Gene	Mean	S.D.	Mean	S.D.	
*tcrac*	2483	1426	1085	846	2.3 F
*igμ*	1395	1207	4259	3549	3.1 M
*bik*	1483	719	4758	1981	3.2 M
*vasa*	2761	1052	1141	889	2.4 F
*gsdf*	1207	664	6761	2807	5.6 M
*inhα*	1961	1319	6321	4884	3.2 M
*amh*	322	110	10648	7167	33.0 M
*cyp19a1a*	4256	3885	422	298	10.1 F

Relative gene expression in males (n = 51) and females (n = 58) is given along with fold change. Expression data include both 15 and 30 dpt samples. Significant enrichment of transcripts in males or females is denoted with M or F letter, respectively. For transcripts and method description, see Methods 5.7. All sex-related effects were significant (ANOVA at p<0.05).

**Table 2:** Correlations (Pearson's *r*) between the relative expressions of the investigated genes.

All variants							
	*igμ*	*bik*	*vasa*	*gsdf*	*inhα*	*amh*	*cyp19a1a*
*tcrac*	**−0.55**	**−0.58**	**F 0.72**	**−0.51**	**−0.26**	**−0.43**	**F 0.32**
*igμ*		**M 0.69**	**−0.59**	**M 0.45**	0.17	**M 0.35**	**−0.39**
*bik*			**−0.57**	**M 0.75**	**M 0.55**	**M 0.71**	**−0.43**
*vasa*				**−0.45**	0.02	**−0.25**	**F 0.52**
*gsdf*					**M 0.76**	**M 0.87**	**−0.47**
*inhα*						**M 0.91**	−0.11
*amh*							**−0.38**

Controls only							
	*igμ*	*bik*	*vasa*	*gsdf*	*inhα*	*amh*	*cyp19a1a*
*tcrac*	**−0.71**	**−0.71**	**F 0.66**	**−0.55**	**−0.43**	**−0.55**	0.16
*igμ*		**M 0.74**	**−0.70**	**M 0.55**	**M 0.36**	**M 0.55**	**−0.41**
*bik*			**−0.61**	**M 0.86**	**M 0.71**	**M 0.81**	**−0.45**
*vasa*				**−0.45**	**−0.09**	−0.29	**F 0.47**
*gsdf*					**M 0.81**	**M 0.87**	**−0.47**
*inhα*						**M 0.95**	−0.23
*amh*							**−0.41**

Treatments only							
	*igμ*	*bik*	*vasa*	*gsdf*	*inhα*	*amh*	*cyp19a1a*
*tcrac*	**−0.51**	**−0.54**	**F 0.73**	**−0.52**	−0.20	**−0.43**	**F 0.40**
*igμ*		**M 0.73**	**−0.56**	**M 0.50**	0.19	**M 0.40**	**−0.40**
*bik*			**−0.58**	**M 0.69**	**M 0.45**	**M 0.67**	**−0.43**
*vasa*				**−0.49**	0.06	**−0.29**	**F 0.54**
*gsdf*					**M 0.71**	**M 0.88**	**−0.49**
*inhα*						**M 0.86**	−0.04
*amh*							**−0.41**

The pattern of correlations is similar in controls (n = 32) and in the treated groups (n = 77). Significant *r*-values (*p*<0.05) are marked with bold font. Significant correlations among male-enriched and female-enriched transcripts are denoted with M or F letters, respectively.

### Effect of treatment on gene expression

Generally, transcript levels showed high variation within all treatments; therefore, the differences in average expression between groups, although frequently considerable, were not always significant. Nevertheless, the effect of the autoimmunization on gene expression was found in all treatments. Certain sex- and time-specific patterns in gene expression were observed. In males, down-regulation in expression of *vasa, inhα*, and *amh* at 30 dpt was observed in all treatments; whereas *igμ* expression at 30 dpt was up-regulated in both sexes (Table 3).

**Table 3:** Expression of marker genes at 15 and 30 days post treatment (dpt).

Treatment	n	Gene							
		*tcrac*	*igu*	*bik*	*vasa*	*gsdf*	*inhα*	*amh*	*cyp19a1a*
**Female 15 dpt**									
Control	7								
Anti-Gsdf-a	3	1.2	−1.4	−1.1	1.0	−2.0	1.2	1.3	1.7
Anti-Gsdf-b	3	−1.5	2.1	1.3	−1.6	−1.4	1.0	−1.1	−1.6
Anti-Gdf9-a	2	1.0	1.7	1.4	−1.6	−1.6	−1.1	1.0	1.4
Anti-Gdf9-b	6	−1.9	**2.6**	**1.8**	**−2.1**	1.5	−1.3	−1.1	−1.2
Anti-Cd205	5	−1.6	**2.9**	**1.7**	**−1.5**	1.0	1.0	1.2	−1.3
**Male 15 dpt**									
Control	6								
Anti-Gsdf-a	3	−1.9	−1.6	−1.3	1.4	1.2	1.7	1.7	−1.3
Anti-Gsdf-b	3	−2.6	**2.5**	−1.3	1.1	−1.1	1.5	1.0	−2.9
Anti-Gdf9-a	3	−3.0	−1.4	1.0	1.7	1.0	1.5	1.5	−3.3
Anti-Gdf9-b	3	1.2	1.2	1.4	−1.5	−1.3	−1.4	−1.1	−1.3
Anti-Cd205	4	−2.1	1.0	−1.3	−2.0	−1.3	−1.4	−1.2	−2.2
**Female 30 dpt**									
Control	9								
Anti-Gsdf-a	4	**1.7**	1.3	−1.3	**1.7**	−1.5	1.9	1.2	1.2
Anti-Gsdf-b	4	1.1	1.2	−1.7	1.2	−1.2	−1.3	1.0	−2.7
Anti-Gdf9-a	4	−1.9	**2.4**	1.2	−1.2	−1.3	1.1	−1.2	1.3
Anti-Gdf9-b	6	−1.4	2.2	−1.2	−1.3	**1.5**	−1.8	**−1.7**	−2.3
Anti-Cd205	6	−2.1	**3.7**	1.4	−1.4	1.3	−1.6	**−1.4**	**−2.9**
**Male 30 dpt**									
Control	10								
Anti-Gsdf-a	2	1.2	**2.5**	−1.2	−1.6	1.2	**−1.9**	**−1.8**	−2.1
Anti-Gsdf-b	4	1.0	1.9	−1.3	−1.7	−1.2	**−1.7**	**−1.9**	−1.3
Anti-Gdf9-a	3	1.0	1.5	−1.2	−1.6	−1.4	**−2.5**	**−1.7**	−1.4
Anti-Gdf9-b	4	1.3	1.1	1.0	**−2.0**	**−1.6**	**−2.8**	**−3.1**	1.1
Anti-Cd205	5	−1.2	2.2	1.2	**−2.2**	**−1.8**	**−3.2**	**−3.1**	−2.2

The relative gene expression is shown as fold change compared to control fish. The values in bold represent a significant difference in expression (ANOVA and LSD test at p<0.05). Different antigens for the same target protein are indicated using –a or –b following the target protein name. All data were normalized using the reference genes *rpl13α* and *ef1α*.

At 15 dpt, in ovaries, up-regulation of *igμ* and *bik* transcripts correlated with down-regulation of *vasa* transcripts in anti-Gsdf-b, anti-Gdf9-a, anti-Gdf9-b, and anti-Cd205 treatments. In the anti-Gdf9-b and anti-Cd205 treatments this relationship was significant for all three genes. In testes, significant up-regulation of *igμ* was observed in the anti-Gsdf-b treatment. No other significant changes in expression were observed. However, down-regulation of *cyp19a1a* transcripts was observed in all treatments (Table 3).

At 30 dpt, many significant changes in gene expression were observed. In ovaries, there was significant up-regulation of *tcrac* and *vasa* expression in the anti-Gsdf-a treatment. In the anti-Gdf9-a treatment, there was significant up-regulation of *igμ* transcripts. The anti-Gdf9-b treatment resulted in significant up-regulation of *gsdf* and down-regulation of *amh* transcripts. In the anti-Cd205 treatment, there was significant up-regulation of *igμ* and down-regulation of *amh* and *cyp19a1a* transcripts. In testes, all treatments resulted in significant down-regulation of *inhα* and *amh* transcripts. The anti-Gdf9-b and anti-Cd205 treatments also resulted in significant down-regulation of *vasa* and *gsdf* transcripts. Significant up-regulation of *igμ* expression was observed in the anti-Gsdf-a treatment (Table 3).

### Effect of treatment on gonadal histology

**Figure 4:**
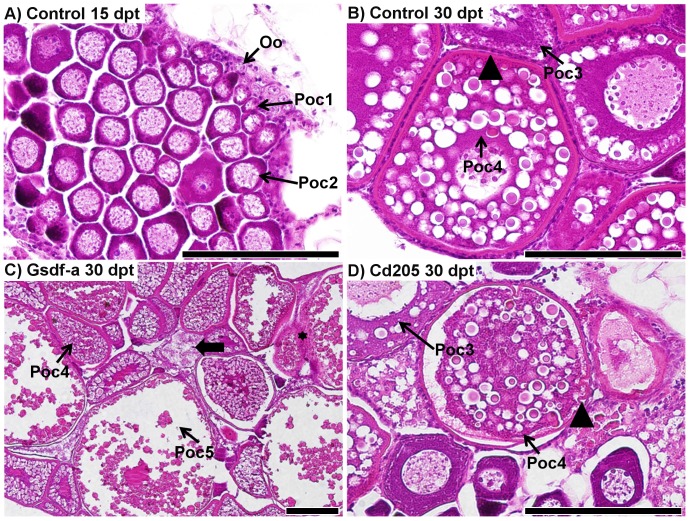
Histological comparison of ovary development in control and experimental fish. A) A representative ovary from a control fish (36.8 mg, 17.2 mm) at 15 days post treatment (dpt); primary oocytes at stage 2 (Poc2) are located predominantly in the center, whereas primary oocytes at stage 1 (Poc1) and oogonia (Oo) are located in the periphery of the ovary. B) A representative ovary from a control fish (216.0 mg, 29.9 mm) at 30 dpt; stage 4 vitellogenic primary oocytes (Poc4) have a thick *zona radiata* (arrowhead) surrounded by an even layer of follicular cells. Stage 3 previtellogenic primary oocytes lack the thick *zona radiata*. C) Ovary from an anti-Gsdf-a treated fish at 30 dpt (326.3 mg, 31.9 mm): stage 4 and 5 (Poc5) primary oocytes weredeveloped. An atretic oocyte is identified by the thick arrow. Granulomatous inflammatory reaction as a consequence of invasion of peritonial cells is identified by the black star. D) Ovary from an anti-Cd205 treated fish at 30 dpt (241.0 mg, 30.5 mm): *zona radiata* is apparently thinner than in controls, and some invaginations are evident (arrowhead), indicating the onset of the atresia process. An irregular follicular layer also indicates atresia (thick arrow). Scalebars represent 100 µm.

At each sampling point, sections of gonads were taken and stained with haematoxylin-eosin. In total, 64 ovary sections were examined: 21 control (7 at 15 dpt, and 14 and 30 dpt), 7 anti-Gsdf-a (2 at 15 dpt and 5 at 30 dpt), 7 anti-Gsdf-b (4 at 15 dpt and 3 at 30 dpt), 11 anti-Gdf9-a (7 at 15 dpt and 4 at 30 dpt), 6 anti-Gdf9-b (30 dpt only), and 12 anti-Cd205 (2 at 15 dpt and 10 at 30 dpt). In controls at 15 dpt, theovaries consisted of a uniform mass of previtellogenic oocytes (Fig. 4A), whereas by 30 dpt, the ovaries often contained previtellogenic and sometimes vitellogenic oocytes with a thick *zona radiata* surrounded by even follicular layers (Fig. 4B). No deviation from this pattern was observed in any control fish. In the treated fish, however, developmental pathology related to *zona radiata* development and oocyte atresia was observed in a number of samples from the anti-Cd205 and anti-Gsdf-a treatments (Fig. 4C, D).

**Figure 5:**
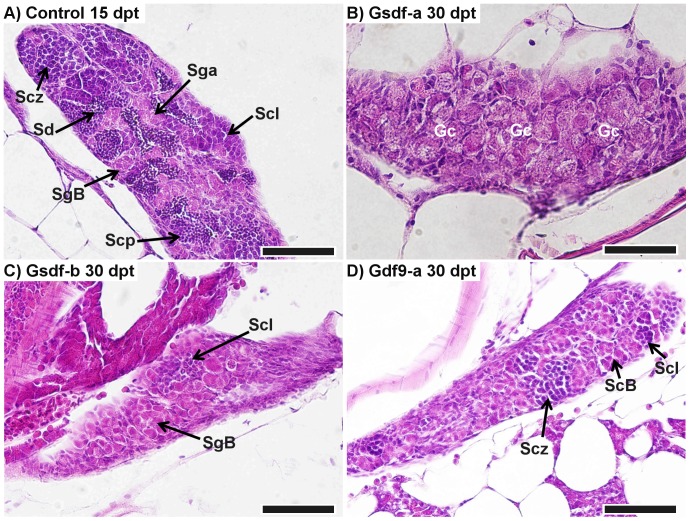
Retardation in testis development. A) Representation of normally developing testis in a control male (78.0 mg, 21.5 mm) at 15 days post treatment (dpt). B) Retarded development in an anti-Gsdf-a treated male at 30 dpt (132.0 mg, 25.5 mm): initial phase of differentiation with only undifferentiated gonocytes visible. C) Testis of an anti-Gsdf-b treated fish at 30 dpt (108.5 mg, 24.0 mm): the testis consisted predominantly of spermatogonia with the start of the spermatocyte phase visible. D) Testis of an anti-Gdf9-a treated fish at 30 dpt (89.4 mg, 22.4 mm): spermatocytes reached the zygotene stage of meiotic prophase. SgA – spermatogonia type A, SgB – spermatogonia type B, Scl – spermatocytes, leptotene of meiotic prophase, Scz – spermatocytes, zygotene of meiotic prophase, Scp – spermatocytes at pachytene stage, Sd – spermatids, Gc – undifferentiated gonocytes. All scalebars represent 50 µm.

Testis sections from 41 males were examined: 9 control (6 at 15 dpt and 3 and 30 dpt), 7 anti-Gsdf-a (2 at 15 dpt and 5 at 30 dpt), 4 anti-Gsdf-b (1 at 15 dpt and 3 at 30 dpt), 11 anti-Gdf9-a (4 at 15 dpt and 7 at 30 dpt), 2 anti-Gdf9-b (30 dpt only), and 8 anti-Cd205 (4 at 15 dpt and 4 at 30 dpt). The treatment effect was primarily through retarded growth and developmental advancement. Control testis at 15 dpt typically consisted of several stages of well-developed clusters of spermatogonial, spermatocyte, and spermatid cells (Fig. 5A). At 30 dpt, testes were further developed and clusters of spermatozoa were visible (Fig. S1A). In contrast, fish from the anti-Gsdf-a and -b treatments at 30 dpt had distinctively smaller and less developed testes (Fig. 5B, C). In anti-Gdf9-a treated fish, some individuals had underdeveloped testes (Fig. 5D); in one extreme case, the testis consisted of undifferentiated gonocytes only (Fig. S1B). There was only a single male examined from the anti-Gdf9-b treatment; its testis was comparatively well developed (Fig. S1C). No obvious developmental retardation was found in the anti-Cd205 treatment (Fig. S1D).

### Effect of treatment on gonadal cell apoptosis

Whole gonads were excised at both 15 and 30 dpt from fish of the *tg*(*vas::egfp*) line. Whole mount immunohistochemistry (IHC) was subsequently performed using a primary antibody specific for pro-apoptotic Bcl2-interacting killer (Bik) protein. In addition, several samples were stained with 4′,6-diamidino-2-phenylindole (DAPI) to visualize associated somatic cells.

**Figure 6:**
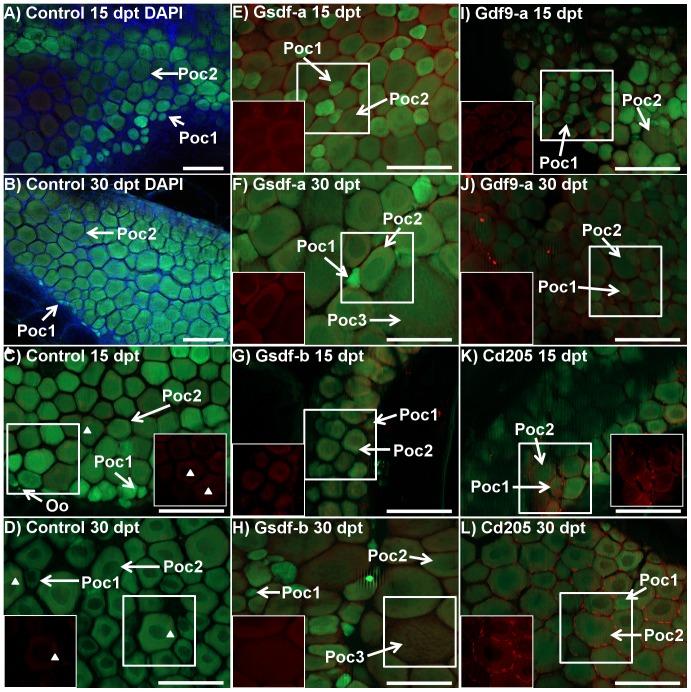
Immunohistochemistry of Bcl2-interacting-killer (Bik) protein in control and treated ovaries of *tg*(*vas::egfp*) line zebrafish. Merged images combine the eGFP filter (green; germ cell lineage), dsRed filter (red; Bik protein), and DAPI filter (blue; somatic cells) under epifluorescent light. A and B) Control fish at 15 (72.3 mg, 20.5 mm) and 30 (108.4 mg, 25.6 mm) days post treatment (dpt), respectively: DAPI staining (blue) identifies somatic cells which fill the space between stage 1 and stage 2 primary oocytes (green) and around the periphery. C and D) Control fish at 15 and 30 dpt, respectively (A: 57.9 mg, 20.0 mm, B: 184.9 mg, 26.9 mm): Ovaries consisted of stage 1 and stage 2 primary oocytes with some oogonia. Very weak Bik signal was detected in some primary oocytes and some unidentified somatic cells (arrowheads) in only some samples. E and F) Anti-Gsdf-a treated fish at 15 and 30 dpt, respectively (E: 80.0 mg, 22.0 mm, F: 126.0 mg, 25.2 mm): Bik signal was detected in stage 2 primary oocytes but not in stage 1 cells, which were undetectable using only the dsRed filter (in box). Some stage 3 previtellogenic oocytes were seen. G and H) Anti-Gsdf-b treated fish at 15 and 30 dpt, respectively (G: 26.8 mg, 15.0 mm, H: 98.7 mg, 23.8 mm): Bik signal was detected in stage 2 primary and stage 3 previtellogenic oocytes. I and J) Anti-Gdf9-a treated fish at 15 and 30 dpt, respectively (I: 48.5 mg, 19.7 mm, J: 73.9 mg, 21.8 mm): the ovary consisted of presumptive stage 1 and 2 primary oocytes with a scattered distribution. Bik signal was detected in oocytes from both stages (in box). K and L) Anti-Cd205 treated fish at 15 and 30 dpt, respectively (K: 72.8 mg, 21.1 mm, L: 190.8 mg, 29.0 mm): Bik signal was detected in both germ cells and supporting somatic cells surrounding stage 1 and stage 2 primary oocytes. The signal in somatic cells was particularly strong. White boxes identify area being shown with dsRED filter only. Poc1 – stage 1 primary oocyte, Poc2 – stage 2 primary oocyte, Oo – oogonia, Poc3 – stage 3 previtellogenic oocyte. All scalebars represent 100 µm.

IHC was performed on 40 ovarian samples: 11 control (5 at 15 dpt, and 6 and 30 dpt), 8 anti-Gsdf-a (3 at 15 dpt and 5 at 30 dpt), 5 anti-Gsdf-b (2 at 15 dpt and 3 at 30 dpt), 9 anti-Gdf9-a (5 at 15 dpt and 4 at 30 dpt), and 7 anti-Cd205 (4 at 15 dpt and 3 at 30 dpt). The general organization of the germ cell and somatic lineages in the ovary is shown in Fig. 6 (A, B). In control ovaries, there was generally a weak signal originating from primary oocytes, but not oogonia (Fig. 6C, D). Also, small, unidentified somatic cells containing Bik at a low level were detected around the primary oocytes at both 15 and 30 dpt. In the treatment groups, Bik signal was clearly stronger and with a more widespread distribution. In both anti-Gsdf-a and –b treatments at both 15 and 30 dpt, Bik was localized in stage 2 primary oocytes, but not in stage 1 oocytes (Fig. 6E → H). At 30 dpt, Bik signal was also detected in stage 3 previtellogenic oocytes. In the anti-Gdf9-a group, Bik protein had a relatively weak signal restricted to stage 1 and stage 2 primary oocytes. No variation in signal was observed between 15 and 30 dpt. However, at both time points the ovaries appeared to develop abnormally, with many smaller primary oocytes in a scattered distribution when compared to control fish (Fig. 6I, J). In the anti-Cd205 treatment, there was a strong Bik signal primarily localized within somatic cells surrounding stage 2 primary oocytes (Fig. 6K, L). This pattern was observed in six of the seven ovaries examined. While the identity of somatic cells could not be confirmed, their distribution suggested supporting follicular cells of the theca and/or granulosa lineage. In primary and previtellogenic oocytes, the Bik signal was similar to that in other treatment groups.

**Figure 7:**
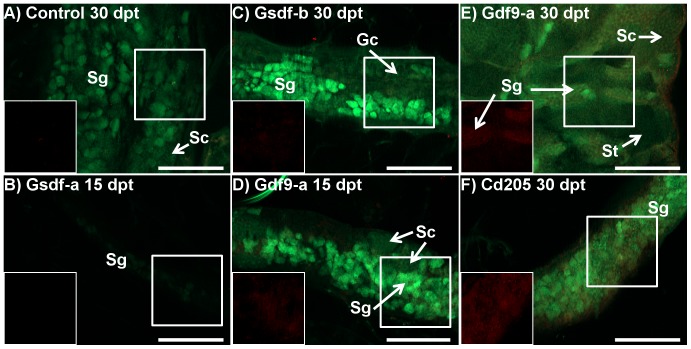
Immunohistochemistry of Bcl2-interacting-killer (Bik) protein in testes of control and treated zebrafish of the *tg*(*vas::egfp*) line. Merged images combine the eGFP filter (green; germ cell lineage), and dsRed filter (red; Bik protein) under epifluorescent light. A) Control fish 30 days post treatment (dpt) (D; 59.3 mg, 21.3 mm): only a few small points of Bik signal were detected. The cell lineage from which the signal originates could not be determined. The testes were developing normally with clusters of spermatogonia (sg) and spermatocytes (sc) visible. B) Anti-Gsdf-a treated fish 15 dpt (27.3 mg, 16.0 mm): The testis was extremely early in development with only a few spermatogonia expressing weak eGFP signal. No Bik signal was detected. C) Anti-Gsdf-b treated fish 30 dpt (86.4 mg, 23.4 mm): Only a very weak Bik signal is detected in the testes. Only presumptive spermatogonia (sg) and undifferentiated gonocytes (gc) were identified. D and E) Anti-Gdf9-a treated fish at 15 and 30 dpt, respectively (D: 46.2 mg, 20.3 mm, E: 138.7 mg, 26.5 mm): Bik signal was localized to clusters of spermatogonia. In contrast, the clusters of spermatocytes (sc) and spermatids (st) did not show Bik signal. F) Anti-CD205 treated fish at 30 dpt (128.4 mg, 25.1 mm): a strong Bik signal was detected throughout the testis. However, resolution was insufficient to determine from which cell types it originated. All scalebars represent 100 µm. White boxes identify area being shown with dsRed filter only.

There were 8 testis samples examined using IHC: 1 control at 30 dpt, 1 anti-Gsdf-a at 15 dpt, 1 anti-Gsdf-b at 30 dpt, 4 anti-Gdf9-a (3 at 15 dpt and 1 at 30 dpt), and 1 anti-Cd205 at 30 dpt. No Bik protein signal was found in the control fish (Fig. 7A). In the anti-Gsdf males, weak or no Bik signal was detected in the testis, which appeared underdeveloped as compared to the control fish (Fig. 7B, C). In the anti-Gdf9-a treatment, weak Bik signal was detected at both 15 and 30 dpt, primarily in spermatogonia (Fig. 7D, E). In the anti-Cd205 treatment, the Bik signal in testis was strong compared to other treatment groups (Fig. 7F). However, it was not possible to determine the cell type the signal originated from.

## Discussion

Immunization of juvenile zebrafish against its own gonadal proteins generally induced an autoimmune response, which consequently affected gonadal development, although the success within and between treatments was variable. The treatment success could be evidenced from differential expression of genes being immune-related or gonadal cell markers, gonad pathology, cell apoptosis, and retarded development. To our knowledge, this is the first demonstration that a peptide-based vaccine against structural or signaling components of a juvenile teleost gonad can affect gonadal development.

### Transcripts encoding target proteins are localized in gonads

We confirmed that transcripts encoding the target proteins were expressed in gonads of zebrafish. *gsdf* expression was localized in the gonadal somatic cells (Figs. 1B and 2B), which is in agreement with the previous report showing *gsdf* expression localized in granulosa and Sertoli cells of zebrafish [23]. Previous studies showed that strong expression of *gdf9* in zebrafish primary growth oocytes (stages 1 and 2) was declining progressively with the follicle development [36]. Our results also demonstrated an inverse relationship between oocyte growth and signal intensity (Fig. 1C). Furthermore, although *gdf9* transcripts were not detected in vitellogenic oocytes, this could be likely due to a lack of sensitivity in the ISH procedure, as previous work showed through RT-PCR that *gdf9* transcripts were present in mature oocytes [36]. Although we found a weak signal of *gdf9* expression in testis, its specific cell localization could not be established (Fig. 2C). ISH for *cd205* transcripts was generally consistent with findings in rainbow trout and bluefin tuna (*Thunnas orientalis*) [34], [35]. In rainbow trout, it was reported that *cd205* transcripts were detected in oogonia, nucleolus-stage oocytes, and type A spermatogonia, whereas in bluefin tuna testis, the transcripts were detected in type A spermatogonia. We detected *cd205* transcripts in stage 1 and 2 primary oocytes, some stage 3 previtellogenic oocytes, as well as spermatogonia (Figs. 1D and 2D).

### Treatment against gonadal proteins induces an immune response

Changes in expression of *tcrac*, which is a mature T-cell receptor marker gene [37], and *igμ*, which is expressed in both secreted and membrane forms of zebrafish Immunoglobulin M (IgM) antibodies [38], have shown the effect of treatments on the immune system. The zebrafish immune system is both morphologically and functionally mature by the age of 4–6 weeks; by this time, mature T and B lymphocytes can be detected, as well as secreted immunoglobulins [37]. The three organs responsible for the majority of immune cell development are the spleen, thymus, and head kidney [37], [39]. Because these organs were not included in the trunk section from which RNA was extracted, we only measured immune cell concentration within the kidney, trunk muscle tissues, digestive system, and gonads.

The T lymphocytes are fundamental to the vertebrate specific immune response. These cells identify antigens through a CD3-associated antigen (Ag)-specific and heterodimeric T cell receptor (TCR) expressed on the cell surface [40]. The majority of mature T-cells displays a αβ TCR which recognizes peptides presented on the surface of antigen-presenting cells (APC) and reside in secondary lymphoid organs. The qPCR data showed that the autoimmunization strategy used in the present study was generally not successful in stimulating increase in T-cell concentration within the gonad (Table 3). Only the anti-Gsdf-a treatment resulted in a significant up-regulation of *tcrac* transcripts in females at 30 dpt. In contrast, while not statistically significant, the other treatments generally showed down-regulation of this marker. In cancer vaccine studies on mice, the use of vaccines with oil-based adjuvant showed that T-cells were far more likely localized in the site of vaccination rather than the sites of tumors, as the injected antigen persisted in oil droplets for extended periods of time [41]. In the current study, peptides were also immersed in an oil-based adjuvant and consequently oil droplets from the vaccines were still observable around the injection site at the time of sampling (not shown). This may explain the general down-regulation of *tcrac* expression, since T-cells could be localized in the site of injection rather than the gonad, and were not included in the sample examined using qPCR. If true, this would be a major limitation of an oil-based adjuvant.

B-cells are lymphocytes in the adaptive immune system, with primary roles in the production of specific antibodies, as well as memory retention of past infections. B-cell activity is performed through B-cell antigen receptor, a multi-component complex on T-cell surfaces comprised of cell surface immunoglobulins, which mediates antigen binding and is responsible for B-lymphocyte maturation, maintenance, activation, and silencing [42], [43]. In the present study, expression of *igμ*, a B-cell marker, was generally up-regulated in the treated males and females (Table 3). B-cells can be present in the kidney as well as the blood; consequently, their specific recruitment to the gonad cannot be confirmed. However, secreted IgM antibodies in the blood would be able to interact with gonadal targets. Much of the elevation in *igμ* concentration was likely due to immunization against keyhole limpet hemocyanin (KLH), which was used as an immunogenic carrier protein in the study. However, the differential expression of *igμ* between different treatment and control groups can be explained by immune response to the specific antigen peptide. Furthermore, transcripts of the apoptosis-promoting gene *bik* were significantly up-regulated in females in anti-Cd205 and anti-Gdf9-b treatments at 15 dpt; it correlated with significant up-regulation of *igμ* expression and could suggest a direct link between increased antibody production and programmed cell death.

### Effect of autoimmunization in gonads is FSH-independent

Follicle stimulating hormone (FSH) is a major regulator of both ovarian and testicular development. Major regulatory pathways are regulated by FSH, such as the Tgfβ pathway, which includes *gdf*9, *gsdf*, *amh* and *inhα*, and is involved in germ cell proliferation and differentiation [44]. Also, FSH is suggested to stimulate Sertoli cell proliferation [45]. In rainbow trout testes, FSH up-regulated *inhα* expression while down-regulated *amh* expression [44]. In the present study, all treatments synergistically resulted in a significant down-regulation of both *inhα* and *amh* transcripts in males at 30 dpt when compared to control fish (Table 3). These findings suggest a mechanism independent of FSH stimulation. There is evidence that the Gsdf role in germ cell proliferation does not involve the FSH regulatory pathway [44]. In the present study, the anti-Gsdf treatments resulted in significant down-regulation in *amh* and *inhα* expression in testes at 30 dpt, indicating that the effect of the treatment on gene expression was not gonadotropin-mediated. Consequently, disturbances in gonadal development were likely the result of direct autoimmunization against gonadal proteins.

### Immunization against germ cell proteins affects somatic supporting cells

Sertoli and granulosa cells derive from a common progenitor cell type and form the first layer of supporting somatic cells surrounding the germ cells in a testis and ovary, respectively [46]. In the gonad, *inhα* and *amh* expression is specific to somatic supporting cells and not germ cells. Inhibitin functions as an endocrine hormone from the gonads to regulate FSH secretion in the pituitary. In zebrafish, *inhα* is predominantly expressed in somatic follicle cells, increasing in expression as folliculogenesis progresses [47]. *Amh* expression is not detected in somatic cells of germ cell-ablated gonads of medaka, which indicates that signaling from germ cells is necessary to sustain *amh* expression in soma [48]. In the present study, both treatments against germ cell-specific proteins (anti-Cd205 and anti-Gdf9) resulted in down-regulation of *inhα* and *amh* in testis and ovary at 30 dpt, while *gsdf* was down-regulated in the testis only (Table 3). This decrease in expression of somatic supporting cell-specific genes could result from a disturbance of soma-germline cross-talk in gonads, caused by an immune response towards the germ cells. Disruption of signaling from germ cells to Sertoli and granulosa cells, affecting further development and functions of Sertoli and granulosa cells, is further supported by follicular histopathology (Fig. 4C, D), intensive apoptosis (Fig. 6K, L), as well as developmental retardation (Fig. 5).

### Effect of anti-Gsdf treatments

Anti-Gsdf treatments resulted in retarded body growth and retarded growth and development of the testes, particularly at 30 dpt (Fig. 5B, C and Fig. 7B, C). Consequently, there was significant down-regulation of *inhα* and *amh* transcripts (Table 3). In contrast, the treatments had little to no effect on female fish at the macroscopic level. Interestingly, histology of some anti-Gsdf-a treated females showed that despite having well-developed ovaries, there were signs of atresia and inflammation (Fig. 4C). Because of the role of *gsdf* in spermatogonial proliferation [21], our results suggest that the treatment interrupted Gsdf signaling, and this suppressed spermatogonia proliferation and subsequent development. It is likely that the treatment was applied too late (6 week-old fish) to hamper ovarian germ cell proliferation, as there were already many primary oocytes present in the ovaries at that time. Since *gsdf* is still strongly expressed in somatic cells surrounding primary oocytes in 7 month-old zebrafish (Fig. 1B), the gene likely retains some function during the ovarian development. However, it is unclear whether the treatment failed to interrupt the signaling, or if the *gsdf* role is not essential for ovarian maturation.

### Effect of the anti-Gdf9 treatments

Treatments against Gdf9 protein resulted in retarded testes development and abnormal ovary development. Furthermore, significant loss in weight, but not in total length was recorded (Fig. 3), which could result from smaller gonads. In testes, the significant down-regulation of *inhα* and *amh* transcripts in both treatments, as well as *vasa* and *gsdf* transcripts in the anti-Gdf9-b treatment (Table 3) was accompanied by retarded development (Fig. 5D). While it has not been confirmed in zebrafish yet, the role of Gdf9 in testes is likely similar to that in ovaries, which is to regulate tight junction integrity between cells of the germ cell lineage and gonadal somatic supporting cells [26], [28]. Interestingly, the strong Bik signal observed in spermatogonia, but not in clusters of spermatocytes and spermatids which had already recruited Sertoli and Leydig cells (Fig. 7E), suggests that an earlier application of the treatment would be more efficient. In femals, significant up-regulation of *igμ* and down-regulation of *vasa* transcripts showed that the effect was strongest at 15 dpt, suggesting that the anti-Gdf9 treatment should be done earlier in females than in males (Table 3). Furthermore, the significant up-regulation of *bik* transcripts at 15 dpt (Table 3), along with localization of Bik protein in primary oocytes (Fig. 6I, J) suggests that preventing Gdf9 signaling between primary oocytes and somatic cells promotes oocyte apoptosis. It is unclear if the treatment had any effect on preventing oocyte maturation, since the fish sampled for histology and IHC were smaller than the control fish, and therefore could not be used for direct comparisons. However, while control ovaries were compact, with a uniform gradient of growth, many anti-Gdf9-treated ovaries appeared abnormal, with a scattered distribution of primary oocytes of various sizes. This could result from impaired Gdf9 signaling, preventing the early stage oocytes from recruiting gonadal somatic cells.

### Effect of the anti-Cd205 treatment

In females, the treatment resulted in reduced growth, elevated expression of immune-related genes, reduced expression of gonadal marker genes, localized signal of pro-apoptotic proteins within the gonad, and irregular gonadal development. The retarded growth at 30 dpt (Fig. 3) could likely result from a reduced ovary mass; as demonstrated in the preliminary experiment, anti-Cd205 treatment resulted in a significant reduction of GSI% in adult females (Table S1). The significant up-regulation of *igμ* and *bik*, combined with significant down-regulation of *vasa* transcripts in 15 dpt ovary (Table 3) suggests a targeted immune response resulting in germ cell ablation. This is supported by histopathological features observed in 30 dpt ovaries (Fig. 4D). The treatment had little to no effect to testes at 15 dpt, whereas significant down-regulation of germ cell (*vasa*) and supporting Sertoli cell (*gsdf*, *inhα*, and *amh*) transcript markers (Table 3) along with a strong Bik signal (Fig. 7F) was found at 30 dpt.

While the function of the Cd205 protein in teleosts has not been studied yet, it may retain some role in antigen presentation to immune cells. In Pacific bluefin tuna, *cd205* transcripts were found not only in the gonad, but also the gills, liver, spleen, and pyloric caeca [35]. In teleosts, the spleen is one of the major lymphoid tissues [49], whereas the gills, spleen and pyloric caeca are important regions for exogenous antigen uptake [50]. In mice, aside from its role in antigen presentation through the MHC pathways, Cd205 is also involved in uptake of “self” antigens from cells undergoing apoptosis and necrosis to induce tolerance to these cells and prevent autoimmunity [33]. Cellular apoptosis is a normal part of gonadal differentiation and development in teleost fishes [17], [18], [19], [51]. Therefore, teleost Cd205 may have an important role in promoting tolerance to self-antigens during early sexual development.

## Conclusions

The present study demonstrates that immunization against specific targets in juvenile zebrafish gonads can induce an autoimmune response and, in consequence, cause disturbances in gonadal development manifested by growth and developmental retardation, intensive apoptosis, atresia, and disturbed transcript production. The treatment effect was likely FSH-independent. Targeting germ cell-specific proteins resulted in disturbed functions of somatic supporting cells, likely through disturbed cross-talk signaling. The anti-Gsdf treatments had little to no effect on females. However, testes were remarkably underdeveloped. The anti-Gdf9 treatments promoted apoptotic signals in testes, and caused abnormal development of ovaries. Finally, anti-Cd205 treatment stimulated a strong immune response in both sexes, resulting in strong pro-apoptotic signals in somatic cells and oocyte atresia. Further optimization of the treatment, including timing, dose, and type of antigen peptide, as well as investigating long-term effects of the treatment, should deliver a highly promising tool to control reproduction in fish.

## Methods

### Fish and husbandry

All husbandry and experimental procedures were performed in accordance with the Norwegian Regulation on Animal Experimentation (The Norwegian Animal Protection Act, No. 73 of 20 December 1974) and were approved by the National Animal Research Authority (Utvalg for forsøk med dyr, forsøksdyrutvalget, Norway) General License for Fish Maintenance and Breeding (Godkjenning av avdeling for forsøksdyr) no. 17. For the preliminary adult zebrafish trial, adults of mixed line were obtained from Febo Norge AS (Oslo, Norway). For the juvenile trial, adult zebrafish from three inbred lines were obtained from the Norwegian School of Veterinary Science (Oslo, Norway). These lines were: AB Tübingen (TAB), serving as a reference line; tg(vasa::vasa-EGFP)zf45 [52], showing GFP expression in the germ cell lineage; and the *nacre* (*-/-*) mutant line, lacking melanophores, thus being transparent. Fish were housed in a zebrafish recirculating system (Aquatic Habitats, Apopka FL, USA) and maintained using standard zebrafish procedures [53]. Water temperature was maintained at 28.0±1.0°C. Broodfish were housed in 10 L tanks at a density of 20 fish/tank with a 1∶1 sex ratio. One month prior to spawning the fish were conditioned with SDS 400 zebrafish specific diet (Special Diet Services, Essex, United Kingdom) and freshly hatched *Artemia* sp. nauplii. Juvenile zebrafish were produced using standard breeding techniques [53]. Post hatch, zebrafish larvae were housed in 1L tanks with fine mesh baffles and restricted water flow. Starting at 5 days post hatch, fish were weaned using SDS 100 zebrafish specific diet (Special Diet Services). From days 14 to 21 they were given a mix of SDS 100 and *Artemia nauplii*. Post day 21 they were only fed *Artemia* nauplii.

### Preliminary trial on adult females

Four experimental vaccines consisted of a synthetic peptide conjugated to the carrier protein keyhole limpet hemocyanin (KLH) (Table S3). Each peptide was chosen from the complete respective amino acid sequence based on predicted antigenicity [54]. The peptides were commercially synthesized at >80% purity (Thermo Fisher Scientific, Ulm, Germany) and conjugated to KLH using a maleimide activated conjugation kit following the manufacturer's protocol (Sigma Aldrich, Oslo, Norway). After conjugation, the protein conjugates were isolated through column chromatography (Sephadex G-25M, Sigma Aldrich) and eluted in phosphate buffered saline solution (PBS). Coupling efficiency was determined by comparing the absorbance at 412 nm from a cysteine standard assay with each peptides cysteine absorbance values before and after conjugation. Final protein concentration was estimated by measuring absorbance at 280 nm. Peptide-KLH conjugates were stored at −20°C until immediately before use. After thawing, the conjugates were diluted in PBS (1.0 mg/mL) and emulsified by vortex at a 1∶1 ratio with Freund's complete adjuvant (FCA, Sigma Aldrich).

In total, 485 adult zebrafish were injected with one of the four treatments or a PBS/FCA control. Because of a limited availability of males, only female zebrafish were considered for this experiment. Each treatment group initially consisted of 105 fish whereas the control group had 65 fish. Prior to immunization, zebrafish were anaesthetized in a bath of 150 mg/L MS-222 (Tricaine; Sigma Aldrich) buffered with 150 mg/L sodium bicarbonate (NaHCO_3_). Fish were considered in surgical anesthesia (stage III) when they became unresponsive to physical stimuli but maintained opercular movement [55]. Fish were removed from the bath and placed on a wet sponge inside a Petri dish. Injections were made intraperitoneally, below the pectoral fin. All injections were 10.0 µL (5.0 µg peptide-KLH) volume. After injection, fish were placed in a recovery tank for 10 min. Injection mortality was 13 out of 485 injected fish. Once recovered, zebrafish were returned to housing tanks.

Zebrafish were sampled immediately prior to immunization. Treated fish were then sampled every 10 days for 30 days. Because of higher mortality, sampling of control fish was only done at 20 dpt. Prior to sampling, fish received an overdose of buffered MS-222 (200 mg/L) and remained in the solution for 10 min following cessation of opercular movement. Upon removal from the bath, the fish were patted dry and weight and fork length measurements were taken. Fish were decapitated before opening the body cavity and the gonads were excised and weighed.

### Autoimmunization vaccine preparation

Peptides for three target proteins (Table 4) were designed based on predicted antigenicity [54]. Six custom peptides (two for each target protein) were synthesized and conjugated to KLH by Thermo Fisher Scientific. Upon arrival, lysophilized peptides were dissolved in PBS to make a stock solution of either 10.0 or 5.0 mg/L, depending on protein solubility. Treatments consisted of either a single antigen (anti-Gdf9 and anti-Gsdf) or, where possible, a combination of both antigens (anti-Cd205) emulsified 1∶1 in FCA. For anti-Gsdf and anti-Gdf9 treatments, peptides were dissolved individually and had a final concentration of 2.5 mg/mL each. Because of high solubility, both Cd205 peptides were combined and retained a final concentration of 2.5 mg/mL.

**Table 4:** Target peptide sequences used as antigens for immunization in the juvenile zebrafish trial.

Target Protein	Accession ID	Treatment	Peptide sequences
Gonadal soma-derived factor	ABZ01522	Anti-Gsdf-a	KSLHL PKEPS NSLSQ C
		Anti-Gsdf-b	SLKNS IHSPP GNSSL C
Growth/differentiation factor 9	NP_001012383	Anti-Gdf9-a	YSFDH NHLSP FSLL C
		Anti-Gdf9-b	QAHKK DIHLL INLT C
Lymphocyte antigen 75	XP_695257	Anti-Cd205	NENDT ESTVR DVYKP C and RRNPN TNNNW EWSDG C

### Immunization

Zebrafish were first injected at 6 weeks post fertilization, with a booster treatment at 15 dpt and final sampling at 30 dpt. The choice of timing for these treatments was based on the window in which the zebrafish can be safely injected (minimum total length of 15 mm; personal observations), have a mature adaptive immune system [37], and have not yet developed a mature gonad [17], [18]. In total, 329 fish (15.5±2.8 mm average ± S.D.) were immunized with one of five vaccines or a PBS/FCA control (Table 5). Fish were anesthetized in a Petri dish filled with tank water containing 50 mg/L MS-222(Sigma Aldrich) buffered with 50 mg/L NaHCO_3_. Each anesthetized fish was first individually photographed and measured for total length using a Zeiss Axio Zoom v.16 microscope. Fish from the tg(vas::egfp) line were also screened for eGFP signal. For injections, fish were transferred to a wet sponge and injected with 2.0 µL of the experimental vaccine using a 50 µL syringe equipped with a 34g needle (Hamilton, Bonaduz, Switzerland). Post injection, fish were transferred to a Petri dish filled with tank water to monitor recovery for 10 min before returning to a housing tank. In total, there were 36 mortalities. For the booster treatment at 15 dpt, the protocol was the same except the vaccination volume was reduced to 1.0 µL and Freund's incomplete adjuvant (FIA) was used as the adjuvant. There was a single mortality from the booster injection.

**Table 5:** Overview of number (n) and lines of fish used in the experiment.

Treatment	Total (n)	Initial length (mm ± S.D.)	15 dpt (n)	30 dpt (n)
Control	29 T	15.7±3.1	13 T	13 T
	15 N		14 N	0 N
	36 V		20 V	13 V
Gsdf-a	13 T	16.0±3.2	6 T	6 T
	7 N		4 N	3 N
	20 V		8 V	12 V
Gsdf-b	15 T	15.3±1.9	6 T	8 T
	3 N		1 N	2 N
	19 V		9 V	9 V
Gdf9-a	19 T	15.6±2.0	6 T	7 T
	25 N		10 N	7 N
	23 V		10 V	11 V
Gdf9-b	19 T	13.9±2.1	9 T	10 T
	5 N			3 N
	18 V			15 V
Cd205	23 T	16.5±2.9	9 T	12 T
	40 V		15 V	21 V

Fish were sampled at 15 and 30 days post-treatment (dpt). Zebrafish lines used were: TAB (T), nacre -/- (N), and tg(vasa::vasa-EGFP) (V).

### Fish sampling

Fish were sampled at 15 and 30 dpt for quantitative real-time PCR (qPCR), histology, and immunohistochemistry (IHC). Furthermore, 7 month-old control fish from the TAB line were sampled for *in situ* hybridization (ISH). Prior to sampling, all fish were euthanized in buffered MS-222 (100 mg/L) so they could be photographed, screened for eGFP signal, and measured for weight and total length (Table 5). Comparisons for sex, weight, and total length were made using the reference TAB line only. The proportion of females:males was 55∶45, not deviating from the expected 50∶50 ratio (Chi-square test).

qPCR analysis was performed solely on TAB line fish. Gonads could not be dissected from such a small fish; therefore, sample sections for analysis contained some fragments of trunk muscle tissue, swim bladder, and kidney, in addition to the gonad. Samples were placed in a 1.5 mL eppendorf tube and snap-frozen in liquid nitrogen. Samples were further stored at −80°C until RNA extraction.

Fish from the *nacre -/-* and *tg*(*vas::egfp*) lines were used for histology and IHC, while the 7 month-old control fish samples for ISH were from the TAB line. The decapitated and truncated body was cut open posterior to the pectoral fins before placing the fish in either Bouin's (for histology and ISH) or 4% paraformaldehyde (PFA, Sigma Aldrich) solution (for IHC). Samples were left in a fixative overnight at 4°C. Fixed samples were then dehydrated in a gradient series of ethanol (from 50% up to 100%).

### *In situ* hybridization

cRNA probes for the genes *cd205* (accession: XM_690165, amplified region 338–2905 nt), *gsdf* (NM_001114668, 33–1302 nt), and *gdf9* (NM_001012383, 134–1956 nt) were produced using methods previously described [56], [57]. The primer set for *gsdf* had previously been verified [20]. Samples were embedded in paraffin wax and systematically sectioned (5–7 µm thick sections) using a rotary microtome (Microm HM355S, MICROM International GmbH, Germany) and adhered to slides coated with 3-aminopropyl triethoxysilane. The hybridization procedure was performed as previously described [56], [57] with the addition of a xylene wash (2×10 min) to de-paraffin the tissue sections prior to rehydration in PBT (PBS with 0.1% Tween 20).

### Quantitative real time PCR

Total RNA was extracted using TRIzol Reagent (Invitrogen, Paisley, U.K.) following the manufacturer's protocol. RNA integrity was first assessed using electrophoresis on a 1% (w/v) agarose gel. Suitable samples were then quantified using NanoDrop ND-1000 (Thermo Scientific, Saven & Werner AS, Kristiansand, Norway). Approximately 1 µg of total RNA was used for cDNA synthesis using the QuantiTect reverse transcription kit (Qiagen, Nydalen, Sweden). All samples were treated with the gDNA wipeout buffer supplied with the QuantiTect reverse transcription kit for 5 min to remove genomic DNA contamination. 20-fold dilutions of cDNA were used for further analysis.

The eight genes selected for qPCR analysis were: T-Cell receptor alpha constant (tcrac), immunoglobulin M heavy chain secretory form (igμ), Bcl2-interacting-killer (bik), vasa (vasa), gonadal somatic cell-derived factor (gsdf), inhibin alpha (inhα), anti-Müllerian hormone (amh), and cytochrome P450 aromatase, gonadal isoform (cyp19a1a). The genes used for reference were beta actin (ß-actin), ribosomal protein L13 alpha (rpl13α) and elongation factor 1 alpha (ef1α). Tcrac and igμ were both established as markers for T and B-cells, respectively [37], [38]. The amplified region of igμ corresponds to the CH3 domain, and is shared by the secreted and membrane forms of the zebrafish immunoglobulin heavy chain protein Igμ [38]. Bik codes for an early pro-apoptosis inducing protein [58], [59]. Vasa is a conserved germ cell marker [60]. Gsdf and inhα are specifically expressed in granulosa and Sertoli cells [20], [47], [61]. Similarly, cyp19a1a is expressed predominantly in granulosa cells, opposite to amh, which is expressed predominantly in Sertoli cells [47]. Specific primers for each target gene for qPCR amplification were either designed manually using Netprimer software (http://www.premierbiosoft.com/netprimer) or taken from literature (Table 6) [47], [62], [63]. When possible, primers were designed to span one intron/exon border to avoid amplification of potential contaminating genomic DNA [64]. All amplified fragments were confirmed as the target gene using Sanger sequencing.

**Table 6:** PCR primers used for quantitative real-time PCR with primer efficiency (E%), co-efficient of determination (R^2^), and amplicon size values.

Gene	Accession ID:	Primer sequence	E%	R^2^	Size
ß-actin*	ENSDART00000055194	Fw: CGAGCTGTCTTCCCATCCA	94.1	0.999	84
		Rv: TCACCAACGTAGCTGTCTTTCTG			
ef1α*	ENSDART00000023156	Fw: CTGGAGGCCAGCTCAAACAT	93.9	0.999	85
		Rv:ATCAAGAAGAGTAGTACCGCTAGCATTAC			
rpl13α*	NM_212784	Fw: TCTGGAGGACTGTAAGAGGTATGC	91.9	0.999	148
		Rv: AGACGCACAATCTTGAGAGCAG			
*tcrac*	AF246178	Fw: CACAACGAGTTCAACATTACCGA	89.8	0.999	194
		Rv: CCAGAAGATGCCCAGTGACAA			
*igμ*	AF281480	Fw: AGCGGAATGATAGCAGGGAGA	89.0	0.999	235
		Rv: TTGGTGAAATGGAATTGTGGA			
*bik*	NM_001045038	Fw: AGATTATGCCGATATTCAGGACG	86.5	0.999	213
		Rv: CCCTCTGTCACATTCAGCCTTC			
*vasa*	NM_131057	Fw: TCAGAGCAACAGGTAATGAGC	90.5	0.998	195
		Rv: CTACAGATGTGGCGACCAGAAC			
*gsdf*	NM_001114668	Fw: GAACGCTCCTGAATCCACAGAC	90.5	0.999	210
		Rv: AATGACTCCCGCAGATGCTC			
inhα**	NM_001045204	Fw: AGCCTCCTCTGCCAGTGTTG	97.7	0.996	188
		Rv: AGCATCAGAAGAGTGGTCAGGTA			
*amh*	AY721604	Fw: TGCTCCTGTTCAGTGTCAATCC	77.5	0.992	180
		Rv: ATGTCTCAACCATCGTCTTCAGT			
*cyp19a1a*	BC163008	Fw: AGATGTCGAGTTAAAGATCCTGCA	93.9	0.999	131
***		Rv: CGACCGGGTGAAAACGTAGA			

Primers previously validated by Tang et al., 2007 [63] (*), Poon et al., 2009 [47] (**), and Jørgensen et al., 2008 [62] (***).

The RT-qPCR was performed on LightCycler 480 (Roche, Mannheim, Germany) using 96-well plates (Roche). SYBR green-based detections were done under the thermal cycle conditions of 95°C for 15 min, followed by 45 cycles of 95°C for 15 s, 63°C for 20 s and 72°C for 10 s. All samples were run in duplicate, together with minus reverse transcriptase, no template and a positive plate control. Five-point standard curves (dilutions 1∶1–1∶81) were used to calculate the efficiency of the PCR reaction. The reaction specificity was evaluated by melting curve analysis. Cycle threshold (*C_T_*) values were determined using the LightCycler 480 software with a level of fluorescence intensity set to one. The reference genes (*ß-actin*, *ef1α, rpl13α*) were examined for data normalization using geNorm [65]. An optimal stability value of 0.486 was obtained using *rpl13α* and *ef1α* and target gene expressions were normalized to relative levels.

### Gonad histology

Sections and slides were prepared as described above in 5.5. Samples were stained with haematoxylin-eosin (HE) in the Robot Slide Stainer (Microm HMS 760X, Thermo Scientific) and mounted using Pertex mounting media (Leica Biosystems). Evaluation and imaging on the samples done by use of the light microscope Olympus BX 51 (Olympus Optical Co. GmbH, Germany) and Olympus Imaging software Cell B. The identification of structures within the zebrafish gonads was done in accordance with the Histology and Histopathology Atlas of the Zebrafish (http://zfishtoxpat.comoj.com).

### Whole mount immunohistochemistry

Whole mount immunohistochemistry was performed to detect Bcl2-interacting-killer (Bik), a pro-apoptotic protein, which is normally suppressed by survival-promoting factors [58]. The procedure was performed as described by Draper (2012) [66] with the following modifications: the primary antibody used was rabbit polyclonal Immunoglobulin G (IgG) specific for anti-zebrafish Bik protein (Anaspec, Belgium). The primary antibody was diluted 1∶150 and administered overnight at 4°C. The secondary antibody used was Alexa Fluor 594 goat anti-rabbit IgG (Life Technologies, Eugene, OR, USA) at a 1∶500 dilution for 5 hours at room temperature. Staining with 4′, 6-diamidino-2-phenylindole (DAPI) was also performed to visualize somatic cells using several washes with a concentration of 2.0 µg/mL. After the completion of the IHC procedure, the gonads were imaged using an AxioZoom V16 microscope equipped with the ApoTome.2 combined with DsRed and eGFP filters. As a positive control to test the procedure, the primary antibody was replaced with an anti-eGFP monoclonal antibody (Invitrogen). Because of a shortage of samples for anti-Gdf9-b treated fish, no whole mount IHC was performed on this treatment group.

### Statistical analyses

Statistical analyses were performed according to Zar (1999) [67]. The data are presented as averages ± standard deviations (S.D.). ANOVA was used to test the effects of treatments on fish weight, length and relative values of gene expression. Homogeneity of variances was tested using Levene's test. Fisher's least significant difference *post-hoc* test was used to investigate differences between groups. Pearson's product-moment correlation coefficient r was used to investigate relationships between gene expressions. Significant effects were considered at *p*<0.05.

## Supporting Information

Figure S1 **Additional testes sections from experimental fish.** A) Representation of normally developing testis in a control male (67.8 mg, 21.5 mm) at 30 days post treatment (dpt). B) Retarded development in an anti-Gdf9-a treated male at 30 dpt (91.0 mg, 22.6 mm): only undifferentiated gonocytes are identified. C) Testis of an anti-Gdf-b treated fish at 30 dpt (78.2 mg, 22.2 mm): clusters of spermatocytes with some spermatids are visible. D) Testis of an anti-Cd205 treated fish at 30 dpt (135.2 mg, 25.1 mm): the testis is well developed with various stages of spermatocytes present. SgA – spermatogonia type A, SgB – spermatogonia type B, Scl – spermatocytes, leptotene of meiotic prophase, Scz – spermatocytes, zygotene of meiotic prophase, Scp – spermatocytes at pachytene stage, Sd – spermatids, Sp – spermatozoa, Gc – undifferentiated gonocytes. All scalebars represent 50 µm.
(TIFF)

Table S1 **Total weight, fork length, and gonadosomatic index (GSI%) of adult zebrafish females immunized with anti-Zona pellucida C (Anti-Zpc), anti-Lymphocyte antigen 75 (Anti-Cd205), anti-Insulin-like growth factor 3 (Anti-Igf3), or anti-Riboflavin carrier protein (Anti-Rcp).**
(DOCX)

Table S2 **Sex-dimorphic gene expression in juvenile zebrafish gonads at 15 and 30 dpt.**
(DOCX)

Table S3 **Target peptide sequences used as antigens for immunization in the adult zebrafish trial.**
(DOCX)

## References

[1] Taranger GL, Carrillo M, Schulz RW, Fontaine P, Zanuy S, et al (2010) Control of puberty in farmed fish. Gen Comp Endocrinol 165:483–515. 19442666 10.1016/j.ygcen.2009.05.004

[2] Cooper DW, Herbert CA (2001) Genetics, biotechnology and population management of over-abundant mammalian wildlife in Australasia. Reprod Fertil Dev 13:451–458. 11999294 10.1071/rd01072

[3] Cooper DW, Larsen E (2006) Immunocontraception of mammalian wildlife: ecological and immunogenetic issues. Reproduction 132:821–828. 17127742 10.1530/REP-06-0037

[4] Karlsen Ø, Holm JC, Kjesbu OS (1995) Effects of periodic starvation on reproductive investment in first-time spawning Atlantic cod (*Gadus morhua* L.). Aquaculture 133:159–170.

[5] Karlsen Ø, Hemre GI, Tveit K, Rosenlund G (2006) Effect of varying levels of macro-nutrients and continuous light on growth, energy deposits and maturation in farmed Atlantic cod (*Gadus morhua* L.). Aquaculture 255:242–254.

[6] McClure CA, Hammell KL, Moore M, Dohoo IR, Burnley H (2007) Risk factors for early sexual maturation in Atlantic salmon in seawater farms in New Brunswick and Nova Scotia, Canada. Aquaculture 272:370–379.

[7] Jørstad KE, van der Meeren T, Paulsen OI, Thomsen T, Thorsen A, et al (2008) “Escapes” of eggs from farmed cod spawning in net pens: recruitment to wild stocks. Rev Fish Sci 16:285–295.

[8] Chen J, Hu W, Zhu Z (2013) Progress in studies of fish reproductive development regulation. Chinese Science Bulletin 58:7–16.

[9] Gui J, Zhu Z (2012) Molecular basis and genetic improvement of economically important traits in aquaculture animals. Chinese Science Bulletin 57:1751–1760.

[10] White YAR, Woods DC, Wood AW (2011) A transgenic zebrafish model of targeted oocyte ablation and de novo oogenesis. Dev Dyn 240:1929–1937. 21761478 10.1002/dvdy.22695

[11] Slanchev K, Stebler J, de la Cueva-Méndez G, Raz E (2005) Development without germ cells: The role of the germ line in zebrafish sex differentiation. Proc Natl Acad Sci U S A 102:4074–4079. 15728735 10.1073/pnas.0407475102 PMC549510

[12] Benfey TJ (1999) The physiology and behavior of triploid fishes. Rev Fish Sci 7:39–67.

[13] Gupta SK, Srinivasan VA, Suman P, Rajan S, Nagendrakumar SB, et al (2011) Contraceptive vaccines based on the zona pellucida glycoproteins for dogs and other wildlife population management. Am J Reprod Immunol 66:51–62. 21501280 10.1111/j.1600-0897.2011.01004.x

[14] Kirkpatrick JF, Lyda RO, Frank KM (2011) Contraceptive vaccines for wildlife: a review. Am J Reprod Immunol 66:40–50. 21501279 10.1111/j.1600-0897.2011.01003.x

[15] Naz DRK (2011) Contraceptive vaccines: success, status, and future perspective. Am J Reprod Immunol 66:2–4. 10.1111/j.1600-0897.2011.00999.x 21645164

[16] Laird L, M., Wilson A, R., Holliday F, G T. (1980) Field trials of a method of induction of autoimmune gonad rejection in Atlantic salmon (*Salmo salar* L.). Reprod Nutr Develop 20:1781–1788. 10.1051/rnd:19801004 7349511

[17] Maack G, Segner H (2003) Morphological development of the gonads in zebrafish. J Fish Biol 62:895–906.

[18] Maack G, Segner H, Tyler CR (2003) Ontogeny of sexual differentiation in different strains of zebrafish (*Danio rerio*). Fish Physiol Biochem 28:125–128.

[19] Rodríguez-Marí A, Cañestro C, BreMiller RA, Nguyen-Johnson A, Asakawa K, et al (2010) Sex reversal in zebrafish *fancl* mutants is caused by Tp53-mediated germ cell apoptosis. PLoS Genet 6:e1001034. 20661450 10.1371/journal.pgen.1001034 PMC2908690

[20] Gautier A, Le Gac F, Lareyre J-J (2011) The gsdf gene locus harbors evolutionary conserved and clustered genes preferentially expressed in fish previtellogenic oocytes. Gene 472:7–17. 21047546 10.1016/j.gene.2010.10.014

[21] Sawatari E, Shikina S, Takeuchi T, Yoshizaki G (2007) A novel transforming growth factor-β superfamily member expressed in gonadal somatic cells enhances primordial germ cell and spermatogonial proliferation in rainbow trout (*Oncorhynchus mykiss*). Dev Biol 301:266–275. 17109839 10.1016/j.ydbio.2006.10.001

[22] Shibata Y, Paul-Prasanth B, Suzuki A, Usami T, Nakamoto M, et al (2010) Expression of gonadal soma derived factor (GSDF) is spatially and temporally correlated with early testicular differentiation in medaka. Gene Expr Patterns 10:283–289. 20601164 10.1016/j.gep.2010.06.005

[23] Gautier A, Sohm F, Joly J-S, Le Gac F, Lareyre J-J (2011) The proximal promoter region of the zebrafish *gsdf* gene is sufficient to mimic the spatio-temporal expression pattern of the endogenous gene in sertoli and granulosa cells. Biol Reprod 85:1240–1251. 21816849 10.1095/biolreprod.111.091892

[24] Paulini F, Melo EO (2011) The role of oocyte-secreted factors GDF9 and BMP15 in follicular development and oogenesis. Reprod Dom Anim 46:354–361. 10.1111/j.1439-0531.2010.01739.x 21198974

[25] Juengel JL, Hudson NL, Heath DA, Smith P, Reader KL, et al (2002) Growth differentiation factor 9 and bone morphogenetic protein 15 are essential for ovarian follicular development in sheep. Biol Reprod 67:1777–1789. 12444053 10.1095/biolreprod.102.007146

[26] Clelland ES, Kelly SP (2011) Exogenous GDF9 but not Activin A, BMP15 or TGFβ alters tight junction protein transcript abundance in zebrafish ovarian follicles. Gen Comp Endocrinol 171:211–217. 21291886 10.1016/j.ygcen.2011.01.009

[27] He Z, Wu Y, Xie J, Wang T, Zhang L, et al (2012) Growth differentiation factor 9 (Gdf9) was localized in the female as well as male germ cells in a protogynous hermaphroditic teleost fish, ricefield eel *Monopterus albus* . Gen Comp Endocrinol 178:355–362. 22732078 10.1016/j.ygcen.2012.06.016

[28] Zhao L, He J, Guo Q, Wen X, Zhang X, et al (2011) Expression of growth differentiation factor 9 (GDF9) and its receptor in adult cat testis. Acta Histochem 113:771–776. 21146857 10.1016/j.acthis.2010.11.005

[29] Dong J, Albertini DF, Nishimori K, Kumar TR, Lu N, et al (1996) Growth differentiation factor-9 is required during early ovarian folliculogenesis. Nature 383:531–535. 8849725 10.1038/383531a0

[30] Liu Z, Chen A, Yang Z, Wei H, Leng X (2012) Molecular characterization of growth differentiation factor 9 and its spatio-temporal expression pattern in gibel carp (*Carassius auratus gibelio*). Mol Biol Rep 39:3863–3870. 21779806 10.1007/s11033-011-1165-8

[31] Heath WR, Belz GT, Behrens GMN, Smith CM, Forehan SP, et al (2004) Cross-presentation, dendritic cell subsets, and the generation of immunity to cellular antigens. Immunol Rev 199:9–26. 15233723 10.1111/j.0105-2896.2004.00142.x

[32] Kato M, Neil TK, Clark GJ, Morris CM, Sorg RV, et al (1998) cDNA cloning of human DEC-205, a putative antigen-uptake receptor on dendritic cells. Immunogenetics 47:442–450. 9553150 10.1007/s002510050381

[33] Shrimpton RE, Butler M, Morel A-S, Eren E, Hue SS, et al (2009) CD205 (DEC-205): A recognition receptor for apoptotic and necrotic self. Mol Immunol 46:1229–1239. 19135256 10.1016/j.molimm.2008.11.016 PMC2680960

[34] Nagasawa K, Shikina S, Takeuchi Y, Yoshizaki G (2010) Lymphocyte antigen 75 (Ly75/CD205) is a surface marker on mitotic germ cells in rainbow trout. Biol Reprod 83:597–606. 20554922 10.1095/biolreprod.109.082081

[35] Nagasawa K, Miwa M, Yazawa R, Morita T, Takeuchi Y, et al (2012) Characterization of lymphocyte antigen 75 (Ly75/CD205) as a potential cell-surface marker on spermatogonia in Pacific bluefin tuna *Thunnus orientalis* . Fish Sci 78:791–800.

[36] Liu L, Ge W (2007) Growth differentiation factor 9 and its spatiotemporal expression and regulation in the zebrafish ovary. Biol Reprod 76:294–302. 17093199 10.1095/biolreprod.106.054668

[37] Lam SH, Chua HL, Gong Z, Lam TJ, Sin YM (2004) Development and maturation of the immune system in zebrafish, *Danio rerio*: a gene expression profiling, in situ hybridization and immunological study. Dev Comp Immunol 28:9–28. 12962979 10.1016/s0145-305x(03)00103-4

[38] Danilova N, Steiner LA (2002) B cells develop in the zebrafish pancreas. Proc Natl Acad Sci U S A 99:13711–13716. 12370418 10.1073/pnas.212515999 PMC129751

[39] Huttenhuis HBT, Huising MO, van der Meulen T, van Oosterhoud CN, Sánchez NA, et al (2005) *Rag* expression identifies B and T cell lymphopoietic tissues during the development of common carp (*Cyprinus carpio*). Dev Comp Immunol 29:1033–1047. 15967501 10.1016/j.dci.2005.03.005

[40] Buonocore F, Castro R, Randelli E, Lefranc M-P, Six A, et al (2012) Diversity, molecular characterization and expression of T cell receptor γ in a teleost fish, the sea bass (*Dicentrarchus labrax*, L). PLoS ONE 7:e47957. 23133531 10.1371/journal.pone.0047957 PMC3485050

[41] Hailemichael Y, Dai Z, Jaffarzad N, Ye Y, Medina MA, et al (2013) Persistent antigen at vaccination sites induces tumor-specific CD8+ T cell sequestration, dysfunction and deletion. Nat Med 19:465–472. 23455713 10.1038/nm.3105 PMC3618499

[42] Hu Y-L, Xiang L-X, Shao J-Z (2010) Identification and characterization of a novel immunoglobulin Z isotype in zebrafish: Implications for a distinct B cell receptor in lower vertebrates. Mol Immunol 47:738–746. 19931913 10.1016/j.molimm.2009.10.010

[43] Miller N, Wilson M, Bengtén E, Stuge T, Warr G, et al (1998) Functional and molecular characterization of teleost leukocytes. Immunol Rev 166:187–197. 9914913 10.1111/j.1600-065x.1998.tb01263.x

[44] Sambroni E, Rolland AD, Lareyre J-J, Le Gac F (2013) Fsh and Lh have common and distinct effects on gene expression in rainbow trout testis. J Mol Endocrinol 50:1–18. 23045716 10.1530/JME-12-0197

[45] Schulz RW, Menting S, Bogerd J, França LR, Vilela DAR, et al (2005) Sertoli cell proliferation in the adult testis - evidence from two fish species belonging to different orders. Biol Reprod 73:891–898. 16000552 10.1095/biolreprod.105.039891

[46] Albrecht KH, Eicher EM (2001) Evidence that Sry is expressed in pre-Sertoli cells and Sertoli and granulosa cells have a common precursor. Dev Biol 240:92–107. 11784049 10.1006/dbio.2001.0438

[47] Poon S-K, So W-K, Yu X, Liu L, Ge W (2009) Characterization of inhibin α subunit (inha) in the zebrafish: evidence for a potential feedback loop between the pituitary and ovary. Reproduction 138:709–719. 19602521 10.1530/REP-09-0198

[48] Kurokawa H, Saito D, Nakamura S, Katoh-Fukui Y, Ohta K, et al (2007) Germ cells are essential for sexual dimorphism in the medaka gonad. Proc Natl Acad Sci U S A 104:16958–16963. 17940041 10.1073/pnas.0609932104 PMC2040408

[49] Press CML, Evensen (1999) The morphology of the immune system in teleost fishes. Fish Shellfish Immunol 9:309–318.

[50] Yang D, Liu Q, Yang M, Wu H, Wang Q, et al (2012) RNA-seq liver transcriptome analysis reveals an activated MHC-I pathway and an inhibited MHC-II pathway at the early stage of vaccine immunization in zebrafish. BMC Genomics 13:319. 22805612 10.1186/1471-2164-13-319 PMC3583171

[51] Janz DM, Van Der Kraak G (1997) Suppression of apoptosis by gonadotropin, 17β-estradiol, and epidermal growth factor in rainbow trout preovulatory ovarian follicles. Gen Comp Endocrinol 105:186–193. 9038251 10.1006/gcen.1996.6820

[52] Krøvel AV, Olsen LC (2002) Expression of a vas::EGFP transgene in primordial germ cells of the zebrafish. Mech Dev 116:141–150. 12128213 10.1016/s0925-4773(02)00154-5

[53] Westerfield M (2000) The zebrafish book. A guide for the laboratory use of zebrafish (*Danio rerio*). Eugene: University of Oregon Press.

[54] Hopp TP, Woods KR (1981) Prediction of protein antigenic determinants from amino acid sequences. Proc Natl Acad Sci U S A 78:3824–3828. 6167991 10.1073/pnas.78.6.3824 PMC319665

[55] Matthews M, Trevarrow B, J M (2002) A virtual tour of the Guide for zebrafish users. Lab Anim 31:34–40. 10.1038/5000140 11923859

[56] Fernandes JMO, MacKenzie MG, Kinghorn JR, Johnston IA (2007) FoxK1 splice variants show developmental stage-specific plasticity of expression with temperature in the tiger pufferfish. J Exp Biol 210:3461–3472. 17873000 10.1242/jeb.009183

[57] Fernandes JMO, MacKenzie MG, Wright PA, Steele SL, Suzuki Y, et al (2006) Myogenin in model pufferfish species: Comparative genomic analysis and thermal plasticity of expression during early development. Comp Biochem Physiol Part D Genomics Proteomics 1:35–45. 20483233 10.1016/j.cbd.2005.09.003

[58] Boyd J, Gallo G, Elangovan B, Houghton A, Malstrom S, et al (1995) Bik, a novel death-inducing protein shares a distinct sequence motif with Bcl-2 family proteins and interacts with viral and cellular survival-promoting proteins. Oncogene 11:1921–1928. 7478623

[59] Chinnadurai G, Vijayalingam S, Rashmi R (0000) BIK, the founding member of the BH3-only family proteins: mechanisms of cell death and role in cancer and pathogenic processes. Oncogene 27:S20–S29. 19641504 10.1038/onc.2009.40 PMC2928562

[60] Yoon C, Kawakami K, Hopkins N (1997) Zebrafish vasa homologue RNA is localized to the cleavage planes of 2- and 4-cell-stage embryos and is expressed in the primordial germ cells. Development 124:3157–3165. 9272956 10.1242/dev.124.16.3157

[61] Rodríguez-Marí A, Yan Y-L, BreMiller RA, Wilson C, Cañestro C, et al (2005) Characterization and expression pattern of zebrafish anti-Müllerian hormone (*amh*) relative to *sox9a*, *sox9b*, and *cyp19a1a*, during gonad development. Gene Expr Patterns 5:655–667. 15939378 10.1016/j.modgep.2005.02.008

[62] Jørgensen A, Morthorst J, Andersen O, Rasmussen L, Bjerregaard P (2008) Expression profiles for six zebrafish genes during gonadal sex differentiation. Reprod Biol Endocrinol 6:1–12. 18590525 10.1186/1477-7827-6-25 PMC2500022

[63] Tang R, Dodd A, Lai D, McNabb WC, Love DR (2007) Validation of zebrafish (*Danio rerio*) reference genes for quantitative real-time RT-PCR normalization. Acta Biochim Biophys Sin 39:384–390. 17492136 10.1111/j.1745-7270.2007.00283.x PMC7110012

[64] Fernandes JMO, Mommens M, Hagen Ø, Babiak I, Solberg C (2008) Selection of suitable reference genes for real-time PCR studies of Atlantic halibut development. Comp Biochem Physiol B Biochem Mol Biol 150:23–32. 18302990 10.1016/j.cbpb.2008.01.003

[65] Vandesompele J, De Preter K, Pattyn F, Poppe B, Van Roy N, et al**.** (2002) Accurate normalization of real-time quantitative RT-PCR data by geometric averaging of multiple internal control genes. Genome Biol 3: research0034.0031 - research0034.0011. 10.1186/gb-2002-3-7-research0034 PMC126239 12184808

[66] Draper B (2012) Identification of oocyte progenitor cells in the zebrafish ovary. In: Mace KA, Braun KM editors. Progenitor Cells: Humana Press. pp.157–165. 10.1007/978-1-61779-980-8_12 22914939

[67] Zar JH (1999) Biostatistical Analysis, 4th edition. New Jersey: Prentice Hall.

