# Supplementary figures and images for: Induced Autoimmunity against Gonadal Proteins Affects Gonadal Development in Juvenile Zebrafish
Source: PLoS One. 2014 Dec 1;9(12):e114209. doi: 10.1371/journal.pone.0114209 (PMC4250200; doi:10.1371/journal.pone.0114209)

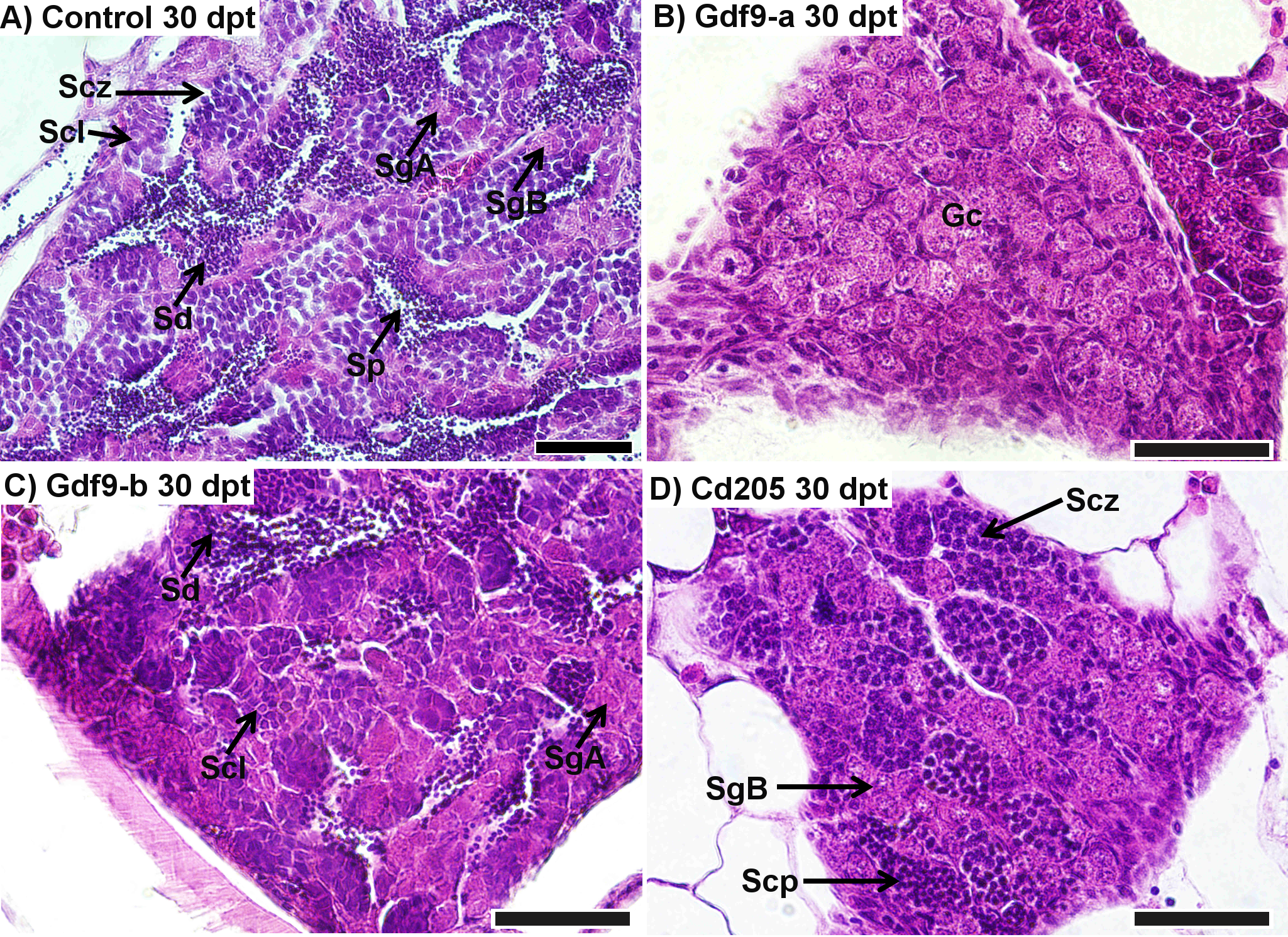

Supplement: Figure S1 — Additional testes sections from experimental fish. A) Representation of normally developing testis in a control male (67.8 mg, 21.5 mm) at 30 days post treatment (dpt). B) Retarded development in an anti-Gdf9-a treated male at 30 dpt (91.0 mg, 22.6 mm): only undifferentiated gonocytes are identified. C) Testis of an anti-Gdf-b treated fish at 30 dpt (78.2 mg, 22.2 mm): clusters of spermatocytes with some spermatids are visible. D) Testis of an anti-Cd205 treated fish at 30 dpt (135.2 mg, 25.1 mm): the testis is well developed with various stages of spermatocytes present. SgA – spermatogonia type A, SgB – spermatogonia type B, Scl – spermatocytes, leptotene of meiotic prophase, Scz – spermatocytes, zygotene of meiotic prophase, Scp – spermatocytes at pachytene stage, Sd – spermatids, Sp – spermatozoa, Gc – undifferentiated gonocytes. All scalebars represent 50 µm. (TIFF) [file pone.0114209.s001.tiff]
