# Supplementary material for: Induced Autoimmunity against Gonadal Proteins Affects Gonadal Development in Juvenile Zebrafish
Source: PLoS One. 2014 Dec 1;9(12):e114209. doi: 10.1371/journal.pone.0114209 (PMC4250200; doi:10.1371/journal.pone.0114209)
Supplement: Table S1 — Total weight, fork length, and gonadosomatic index (GSI%) of adult zebrafish females immunized with anti-Zona pellucida C (Anti-Zpc), anti-Lymphocyte antigen 75 (Anti-Cd205), anti-Insulin-like growth factor 3 (Anti-Igf3), or anti-Riboflavin carrier protein (Anti-Rcp). (DOCX) [file pone.0114209.s002.docx]

Table S1. Total weight, fork length, and gonadosomatic index (GSI%) of adult zebrafish females immunized with anti-Zona pellucida C (Anti-Zpc), anti-Lymphocyte antigen 75 (Anti-Cd205), anti-Insulin-like growth factor 3 (Anti-Igf3), or anti-Riboflavin carrier protein (Anti-Rcp).

|  | Weight (g) | | | Fork length (mm) | | | GSI (%) | | |
| --- | --- | --- | --- | --- | --- | --- | --- | --- | --- |
|  | N | Mean | S.D. | N | Mean | S.D. | N | Mean | S.D. |
| Control* | 32 | 0.98 | 0.12 | 32 | 42.8 | 2.5 | 26 | 12.3 | 3.5 |
| Control 20 dpt* | 18 | 1.01 | 0.21 | 18 | 43.2 | 2.7 | 18 | 13.8 | 4.7 |
| Anti-Zpc 10 dpt | 29 | **0.90** | **0.13** | 29 | 42.0 | 1.5 | 29 | **8.2** | **3.3** |
| Anti- Zpc 20 dpt | 28 | 0.95 | 0.13 | 28 | 43.0 | 2.1 | 28 | **9.3** | **2.6** |
| Anti- Zpc 30 dpt | 20 | **1.11** | **0.17** | 20 | 43.6 | 2.0 | 20 | 13.4 | 3.7 |
| Anti-CD205 10 dpt | 27 | **0.89** | **0.12** | 27 | 41.9 | 1.6 | 26 | **9.0** | **3.7** |
| Anti-CD205 20 dpt | 28 | 0.96 | 0.17 | 28 | 43.3 | 2.4 | 27 | 10.6 | 2.6 |
| Anti-CD205 30 dpt | 27 | 1.02 | 0.19 | 27 | 43.1 | 1.8 | 27 | **10.1** | **2.4** |
| Anti-Igf3 10 dpt | 20 | 1.01 | 0.14 | 20 | 43.8 | 2.2 | 20 | 12.5 | 4.7 |
| Anti- Igf 3 20 dpt | 25 | 0.97 | 0.12 | 25 | 43.1 | 2.3 | 25 | 12.9 | 4.2 |
| Anti- Igf 3 30 dpt | 28 | **1.13** | **0.17** | 28 | 44.1 | 2.0 | 22 | 14.4 | 4.7 |
| Anti-Rcp 10 dpt | 17 | 0.95 | 0.16 | 17 | 42.6 | 2.2 | 17 | 12.2 | 3.5 |
| Anti- Rcp 20 dpt | 16 | 1.00 | 0.19 | 16 | 42.5 | 2.1 | 16 | 12.8 | 3.6 |
| Anti- Rcp 30 dpt | 17 | 1.04 | 0.15 | 17 | 43.3 | 1.7 | 17 | 14.6 | 3.6 |

Sampling was performed at day 0 (Control), 10, 20 and 30 days post-treatment (dpt). * Controls for 10dpt and 30 dpt are missing because of disease outbreak; however, fish were adult and not growing significantly in the course of experiment. N = number of fish per treatment; S.D. = standard deviation. Bold font indicates values significantly different from controls (ANOVA,

*p* < 0.05).
