# Supplementary material for: Induced Autoimmunity against Gonadal Proteins Affects Gonadal Development in Juvenile Zebrafish
Source: PLoS One. 2014 Dec 1;9(12):e114209. doi: 10.1371/journal.pone.0114209 (PMC4250200; doi:10.1371/journal.pone.0114209)
Supplement: Table S2 — Sex-dimorphic gene expression in juvenile zebrafish gonads at 15 and 30 dpt. (DOCX) [file pone.0114209.s003.docx]

Table S2. Sex-dimorphic gene expression in juvenile zebrafish gonads at 15 dpt (males, n=21; females, n=27) and at 30 dpt (males, n=29; females, n=32).

| **15 dpt** |  |  |  |  |  |
| --- | --- | --- | --- | --- | --- |
|  | **female** |  | **male** |  |  |
|  | mean | S.D. | mean | S.D. | fold change |
| *tcrac* | 2396 | 860 | 1068 | 656 | **2.2 F** |
| *igµ* | 1591 | 1203 | 5376 | 3153 | **3.2 M** |
| *bik* | 1477 | 619 | 4571 | 2140 | **3.1 M** |
| *vasa* | 2475 | 709 | 877 | 708 | **2.8 F** |
| *gsdf* | 1367 | 524 | 5866 | 2011 | **4.3 M** |
| *inhα* | 1581 | 691 | 4355 | 3340 | **2.8 M** |
| *amh* | 306 | 99 | 7814 | 4169 | **25.6 M** |
| *cyp19a1a* | 3325 | 2415 | 396 | 264 | **8.4 F** |

| **30 dpt** |  |  |  |  |  |
| --- | --- | --- | --- | --- | --- |
|  | **female** |  | **male** |  |  |
|  | mean | S.D. | mean | S.D. | fold change |
| *tcrac* | 2358 | 1186 | 1045 | 658 | **2.3 F** |
| *igµ* | 1317 | 729 | 4245 | 2183 | **3.1 M** |
| *bik* | 1576 | 618 | 4757 | 1270 | **3.0 M** |
| *vasa* | 2776 | 702 | 1245 | 625 | **2.2 F** |
| *gsdf* | 942 | 305 | 7804 | 2040 | **8.3 M** |
| *inhα* | 2207 | 1273 | 7402 | 3400 | **3.4 M** |
| *amh* | 314 | 78 | 12170 | 5252 | **38.8 M** |
| *cyp19a1a* | 5158 | 3547 | 431 | 191 | **12.0 F** |

Relative gene expression is given along with fold change. Expression data are given for 15 and 30 dpt samples separately. Enrichment of transcripts in males or females is denoted with M or F letter, respectively. For transcripts and method description, see Methods 5.7. Fold changes between females and males were significant (ANOVA at p < 0.05).
