# Supplementary material for: Induced Autoimmunity against Gonadal Proteins Affects Gonadal Development in Juvenile Zebrafish
Source: PLoS One. 2014 Dec 1;9(12):e114209. doi: 10.1371/journal.pone.0114209 (PMC4250200; doi:10.1371/journal.pone.0114209)
Supplement: Table S3 — Target peptide sequences used as antigens for immunization in the adult zebrafish trial. (DOCX) [file pone.0114209.s004.docx]

Table S3. Target peptide sequences used as antigens for immunization in the adult zebrafish trial.

| Target Protein | Abbreviation | Accession ID | Peptide sequence |
| --- | --- | --- | --- |
| Lymphocyte antigen 75 | Cd205 | XP_695257 | FKTDG FEDDG DDSEE C |
| Insulin-like growth factor 3 | Igf3 | NP_001108522 | LYCAK SKKVR RDVPA C |
| Riboflavin carrier protein | Rcp | NP_001018566 | RVQEG DPEEL DTTKS C |
| Zona pellucida glycoprotein C | Zpc | CAH69084 | ASKFL PRVKD DKLRF C |
